# Noise-induced differences in the complexity of spoken language

**DOI:** 10.1177/17470218221124869

**Published:** 2022-10-06

**Authors:** Catherine T Pham, Elisabeth A Karuza

**Affiliations:** Department of Psychology, The Pennsylvania State University, University Park, PA, USA

**Keywords:** Cognitive control, complexity, language production, speech, syntax

## Abstract

Although speaking in noisy environments is a common occurrence, few studies have investigated how noise affects language production beyond the acoustic level. In seeking to differentiate between speaker- and listener-oriented modifications, this study examines the effect of noise on the complexity of language production and examines whether cognitive control predicts noise-induced modifications. Participants completed a picture description task via videoconferencing software while both the speaker (the participant) and listener (the experimenter) were exposed to multi-talker babble. Speakers produced fewer T-units, clauses, and words as well as fewer, but longer, unfilled pauses in noise. The degree of reduction in number of clauses, words, and unfilled pauses was significantly associated with weaker cognitive control. Thus, we consider these modifications to be speaker-oriented, driven by the distracting nature of noise. However, participants also produced fewer filled pauses and mazes in noise. These modifications were not significantly correlated with cognitive control, and they diverge from prior work demonstrating that speakers tend to produce *more* disfluencies when they alone shoulder the burden of a noisy environment. This pattern of results suggests that speakers may alter their speech to alleviate cognitive burden on themselves as well as to facilitate comprehension for their listener.

Spoken language is typically produced in noisy environments. Consider the types of noise a person may encounter in their day-to-day life: a quiet library (30 dB), the humming of a refrigerator (40–50 dB), a classroom full of children (60–90 dB), busy traffic (70–85 dB), or a bustling restaurant (80–90 dB) (Erickson & Newman, 2017). Rarely do we experience true silence, yet psycholinguistic research has largely focused on examining speech production under optimal, relatively silent conditions. The studies that have investigated the effect of noise on speech production have primarily done so at the acoustic level, centring on speakers’ tendencies to increase the loudness of their speech in noisy environments (Lombard, 1911; see also Castellanos et al., 1996; Junqua, 1993; Summers et al., 1988). Although it is clear that speakers alter the *acoustic properties* of their speech in response to noise, whether they alter the *syntactic complexity* of their speech remains an open question.

The challenges speakers face in noisy environments are twofold: speakers must contend with (1) the distracting nature of noise coupled with (2) knowledge of the transmission degradation of the speech signal, which results in their listener engaging in more effortful processing. Previous research indicates that sentence comprehension is more difficult in the presence of background noise, especially as structures become more syntactically complex (e.g., Carroll & Ruigendijk, 2013; Dillon, 1995; Wendt et al., 2015, 2016). Recent work also suggests that complex sentence structures are more difficult to produce than simpler structures (Santi et al., 2019; Scontras et al., 2015). In light of these findings, we predicted that speakers would reduce the complexity of their speech under noisy conditions, but we also considered that the impetus for these changes might not necessarily be speaker-oriented. A reduction in complexity may also be a means of mitigating the listener’s burden of comprehension under adverse conditions. To our knowledge, few studies have directly investigated such higher-level changes, despite the pervasiveness of noise in everyday life. As detailed below, those that have (Harmon et al., 2021; Kemper et al., 2003), were best positioned to examine the demands placed on the speaker, utilising paradigms that minimised the burden of the listener under effortful listening conditions. To address this gap, the present study, conducted online via videoconferencing software, compared speech produced (and received by the listener) in noise versus (relative) silence during a picture description task, further examining whether patterns of speech produced in noise might be modulated by individual differences in cognitive control as a means of beginning to differentiate speaker- versus listener-oriented modifications.

## The effect of noise on the acoustic properties of speech production

It is well attested that speakers alter the acoustic properties of their speech in the presence of noise (e.g., Castellanos et al., 1996; Junqua, 1993; Lombard, 1911; Pisoni et al., 1985; Summers et al., 1988). The *Lombard effect* refers to an automatic increase in vocal effort when speaking in noise, resulting in perceptually louder speech. Noise-induced acoustic modifications include changes in amplitude, formant frequencies, intensity, and vowel duration (for a review, see Junqua, 1996). Although the Lombard effect is involuntary, the type of background noise modulates the degree to which speakers alter certain properties of their speech (e.g., Garnier et al., 2006; Junqua, 1994; Lu & Cooke, 2008). For example, speakers exhibit a greater increase in vowel duration when speaking in multi-talker babble relative to white noise (Junqua, 1994). Lombard effects are also modulated by whether or not individuals are engaged in a communicative task. Garnier et al. (2010) reported that noise-induced modifications were amplified when participants completed a task with a speech partner compared with when they did so alone, suggesting that, to some extent, speakers produce Lombard speech to facilitate communication.

## The effect of noise on the comprehension of syntactically complex structures

Generally, the ways in which noise affects syntactic complexity have been under-studied within the realm of speech *production*, but several studies have examined how noise affects the *processing* of complex structures (Carroll & Ruigendijk, 2013; Dillon, 1995; Hyönä & Ekholm, 2016; Obleser et al., 2011; Wendt et al., 2015, 2016; Yampolsky et al., 2002). Yampolsky et al. (2002) did not observe an interaction between the presence of speech-shaped noise and syntactic complexity when examining listening durations during an auditory moving window task. However, offline plausibility judgements of complex sentences were influenced by noise. More recently, Carroll and Ruigendijk (2013) also investigated the effect of speech-shaped noise on the comprehension of German sentences of varying complexity using a word monitoring task. Reaction times were measured at syntactically critical points in each sentence. The processing of more complex, non-canonical object–verb–subject (OVS) structures was more affected by noise than less complex, canonical subject–verb–object (SVO) structures, suggesting that cognitive demands associated with processing syntactically complex structures are exacerbated in noise. Dillon (1995) examined the effect of multi-talker babble on the processing of English sentences of varying levels of syntactic complexity. During an object manipulation task, participants were instructed to act out sentences with figurines. Observing a greater number of errors and increased response latencies, Dillon reported that complex sentences were more difficult to comprehend, particularly at higher levels of background noise.

Taken together, these studies, ordered in terms of noise complexity, provide evidence that the difficulties associated with processing more complex sentences are heightened under adverse listening conditions; however, this effect is influenced by both the types of syntactic structures being examined and the types of background noise being presented. Informational masking is driven by features of the background noise that may tax the listener’s cognitive resources, including competition for attention, interference from a known language, and higher cognitive load (Mattys et al., 2009). Speech-shaped noise, which generally does not induce informational masking, seems to have weaker effects of the processing of complex syntactic structures compared with multi-talker babble. However, Hyönä and Ekholm (2016) reported that intelligible background speech (which has greater potential for informational masking compared with speech-shaped noise and babble) only exacerbated complexity effects on processing when it consisted of scrambled, instead of coherent, background speech. This scrambled speech was created by randomising the word order of a speech sample and presenting this list of words with normal speech intonation. Syntactic complexity effects were not affected by meaningful, coherent (i.e., semantically and syntactically intact) background speech. Thus, it seems that the effect of noise on the processing of complex structures not only depends on the complexity of the noise and its potential for informational masking, but also the predictability of any linguistic content present in the noise.

## The effect of noise on higher-level properties of speech production

Within the realm of production, several studies have investigated how the presence of background noise affects turn-taking behaviour in conversational contexts. In general, speaking in noisy conditions leads to longer utterance durations (Beechey et al., 2018; Sørensen et al., 2020, 2021; Watson et al., 2020). Potentially, this lengthening allows speakers more time for speech planning under more demanding conditions. However, Hadley et al. (2019) reported shorter utterance durations as noise levels increased. They proposed this unexpected reduction may be attributed to either their use of a relatively free conversation task compared with more goal-oriented tasks (e.g., Diapix task, map task) or use of more variable background noise (fluctuating from 54 to 78 dB) compared with the constant levels found in other studies. Relatedly, other work has examined how turn-taking in one conversation is affected by the presence of a simultaneous conversation between another pair of speakers, reporting decreased overlap between interlocutors (Aubanel et al., 2012) and longer inter-turn pauses (i.e., gaps between when one speaker stops talking and the other speaker begins) (Aubanel et al., 2011). From the perspective of the listener, longer inter-turn pauses could be beneficial, allowing more time for comprehension. However, Hadley et al. (2019) reported shorter inter-turn pauses in the presence of a competing conversation. Thus, it remains unclear whether noise-induced modifications in inter-turn pause duration occur for the listener’s benefit.

With the exception of studies examining turn-taking, the handful of studies that have studied the effect of noise on speech production beyond the acoustic level were not positioned, due to their experimental set-up, to examine the role of the listener. The idea that speakers (sometimes) alter their production to accommodate the needs of their interlocutor is referred to as *audience design* (Clark & Murphy, 1982). The role of the listener is an important consideration given that the Lombard effect is more pronounced in communicative contexts (e.g., Garnier et al., 2010). Furthermore, evidence suggests that speakers strategically alter their speech depending on the type of adverse listening condition experienced by their interlocutor (e.g., Hazan & Baker, 2011). At the syntactic level, audience design would entail the speaker’s implicit or explicit understanding that complex grammatical structures are more difficult to comprehend. As such, speakers may produce simpler structures to lessen the burden on the listener in noise. The studies presented below have solely examined contexts in which the speaker had to contend with the background noise, but the listener did not. Thus, these studies, although informative, cannot directly address noise-induced changes related to the speaker’s knowledge of the transmission degradation of the speech signal. Kemper and colleagues (2003) examined how the presence of background noise affected fluency, syntactic complexity, and propositional content (i.e., amount of information conveyed) in speech production in younger and older adults. Participants answered primarily autobiographical questions while ignoring speech (a recording of an individual reading semantically anomalous sentences) and while ignoring noise (a recording of ambient cafeteria noise). Participants exhibited decreased speech rates while speaking in noise, decreased syntactic complexity, and decreased propositional content. Differences were generally greater when ignoring speech than ignoring ambient cafeteria noise, indicating that the amount of linguistic interference present in the background noise affects language production.

Harmon et al. (2021) also investigated the effect of noise on the higher-level properties of speech production, specifically probing the effect of five different types of background noise containing varying degrees of linguistic interference (a two-person debate, dialogue from a movie, contemporary music with English lyrics, classical music with Latin vocals, and pink noise). Participants answered prompts that they selected for themselves from a list of available options. Notably, participants exhibited increased disfluencies, defined as false starts and repetitions in their study, across all noise conditions (with the exception of pink noise) and fewer unfilled pauses across all noise conditions (with the exception of classical noise). Overall, it seems that the amount of linguistic interference present in noise affected error production, specifically lexical-phonological and morpho-syntactic errors. Speakers produced significantly fewer grammatically correct words in the debate noise condition relative to the silent condition. Although effects did not reach the level of statistical significance, greater lexical errors were observed for both the movie noise condition and the debate noise condition compared with silence.

Taken together, these two studies (Harmon et al., 2021; Kemper et al., 2003) provide evidence that noise does affect non-acoustic properties of speech production. Notably, in both studies, background noise was presented to participants through headphones, so the experimenter (the listener) was not privy to the noise. Building on Harmon et al.’s (2021) proposal that a subset of noise-induced modifications may represent interference effects due to background noise while other modifications may reflect compensatory strategies employed to facilitate the listener’s comprehension, the current study further explored the possibility of listener-oriented changes by ensuring that both the speaker and the listener were exposed to background noise. Moreover, a benefit of the present study’s method of data collection is that simultaneous exposure to background noise could occur without sacrificing the quality of the audio recording. As further detailed below, we also included measures of cognitive control as a means of exploring potential forces behind noise-induced differences in complexity. Given that speaking in noise entails ignoring distracting auditory input, speakers with greater cognitive control may be better able to contend with the distracting nature of noise and exhibit fewer differences between speech produced in noise versus silence.

## The present study

The present study investigated whether speakers alter the syntactic complexity of their speech in the presence of background noise during a picture description task administered via videoconferencing software. Our measures of syntactic complexity were divided into three categories: ratio-based measures, measures of quantity. and measures of disfluencies and errors. Our ratio-based measures (mean length of T-unit [MLTU] and clausal density [CD]) are arguably the most commonly utilised metrics of syntactic complexity (Jagaiah et al., 2020). Both MLTU and CD are utilised in the fields of communication sciences and disorders (e.g., Nippold et al., 2014) and applied second language acquisition (e.g., Lu, 2011), with higher MLTU and CD considered to be reflective of greater syntactic complexity. Measures of quantity (number of T-units, clauses, and words) were also examined. Although these are not traditionally considered as measures of complexity per se, they capture whether people generally spoke less in noise condition, a reasonable expectation given the presumed cognitive burden of speaking in noise. Syntactic complexity can also be operationalised in terms of the effort required to produce or comprehend a particular structure. Disfluencies most often appear where greater demand on utterance planning emerges, and the rate of disfluencies increases as utterance planning difficulty increases (Bortfeld et al., 2001). Complex structures have been argued to require more cognitive resources to plan (MacDonald, 2013) and produce (Santi et al., 2019; Scontras et al., 2015). Thus, increased rates of disfluencies might be attributed to increases in the production of complex syntactic structures in silence, increased effort associated with utterance planning in noisy environments, or some combination of these two factors. We predicted that speech produced in the context of noise would be less syntactically complex than speech produced in silence, as reflected by lower MLTU and CD and reduced quantity of speech (i.e., fewer T-units, clauses, and words). We also predicted that these reductions in complexity would be accompanied by increases in disfluencies and errors due to challenges in utterance planning under noisy conditions.

As previewed above, a key facet of the present study is that it also explored the extent to which noise-induced changes in syntactic complexity were modulated by individual differences in cognitive control as a means of beginning to tease apart speaker-oriented versus listener-oriented modifications. Cognitive control, a key component of executive function, refers to processes that regulate thoughts and behaviour. It has been subcategorised into three main types: shifting between tasks, monitoring and updating working memory, and inhibiting prepotent responses (Miyake et al., 2000). Of most relevance to the current work, evidence from both behavioural and neuroimaging studies have demonstrated that language production involves inhibitory control (e.g., Bourguignon et al., 2018; Nozari & Omaki, 2022; Shao et al., 2013, 2014; Sikora et al., 2016; Zhang et al., 2018). To produce fluent and grammatically correct speech, speakers must suppress task-irrelevant stimuli and engage in a monitoring process that has been argued to involve some degree of cognitive control (e.g., Piai et al., 2013); the effort associated with this suppression is likely to be particularly great when speakers grapple with background noise. Accommodating a listener who is simultaneously contending with noise can also be a demanding task, especially if the needs of the speaker and listener do not align. Cognitive control has been implicated in speakers’ ability to engage in perspective-taking when producing referential expressions (Brown-Schmidt, 2009; Long et al., 2018; Saryazdi & Chambers, 2021; Trude & Nozari, 2017; Wardlow, 2013). However, little work has examined the role of cognitive control under adverse speaking conditions.

Three tasks were selected to assess different aspects of cognitive control: an AX-Continuous Performance Task (AX-CPT; Braver et al., 2001), a Flanker task (Eriksen & Eriksen, 1974), and a counting Stroop task (Bush et al., 2006). The AX-CPT allows for the examination of proactive control and reactive control. Proactive control involves anticipating and preventing interference before it can occur whereas reactive control involves detecting and resolving interference after it occurs (Braver, 2012). Within the context of speaking in noise, speakers may implement proactive control, preparing to alter their speech in anticipation of noise-induced disruptions in utterance planning, or they may implement reactive control, altering their speech only when noise disrupts their utterance planning process. From an audience design perspective, as described by Long and colleagues (2020), if the speaker’s goal is to produce minimal expressions, the speaker may engage in a proactive control strategy that focuses on the goal, leading to the inhibition of potentially relevant information (e.g., the colour of an object). However, in certain contexts, the speaker may engage in reactive control and flexibly adapt to relevant contextual cues to produce more detailed descriptions if doing so would benefit the listener (e.g., describing the colour of an object if it facilitates the listener’s visual search of a scene). The Flanker and counting Stroop tasks involve overcoming interference effects and reflect the suppression of task-irrelevant stimuli (i.e., noise). The counting Stroop task involves linguistic stimuli and has been argued to involve a more prepotent incorrect response compared with the Flanker (Nee et al., 2007). Thus, by examining performance on these two tasks, we intended to compare linguistic and non-linguistic cognitive control. We predicted individuals with greater cognitive control would be less susceptible to the additional cognitive burdens of speaking in noise, exhibiting fewer differences in complexity for speech produced in noise relative to silence.

## Method

### Participants

In all, 62 participants recruited from the Pennsylvania State University’s Psychology SONA subject pool participated in this study (56 females, 6 males; age: *M* = 18.90 years, *SD* = 1.18 years). In exchange for their participation, participants received one research credit. All participants were monolingual native English speakers with self-reported normal or corrected-to-normal vision and hearing. Overall, data from five participants were excluded: three due to the presence of extraneous background noise noticed by the experimenter, one due to the completion of the study while wearing a face mask, and one due to audio recording problems; 57 participants were included in the speech production analyses.

An a priori power analysis indicated that a sample size of 34 participants would be sufficient to detect a medium effect (*d* = 0.5) for a within-group comparison of speech produced in noise compared with silence (using a Type I error rate of 0.05 and a Type II of 0.2). We are justified in selecting this effect size given that related work has revealed large effect sizes when examining acoustic–phonetic changes in noisy relative to silent conditions (e.g., Vogel et al., 2014; *d* = 1.53)—a more conservative, medium effect size allowed for the possibility that changes in syntactic complexity might be less pronounced. In addition, prior work by Vuong and Martin (2014) found a significant correlation (*r* = .37) between cognitive control and comprehension of garden path sentences, which are typically considered as highly complex. An a priori power analysis indicated that a sample size of 55 participants would be sufficient for similar planned correlational analyses in the present study. Thus, our final sample size of 57 participants was adequate for our planned analyses.

### Stimuli

#### Picture description task

**Figure 1.**
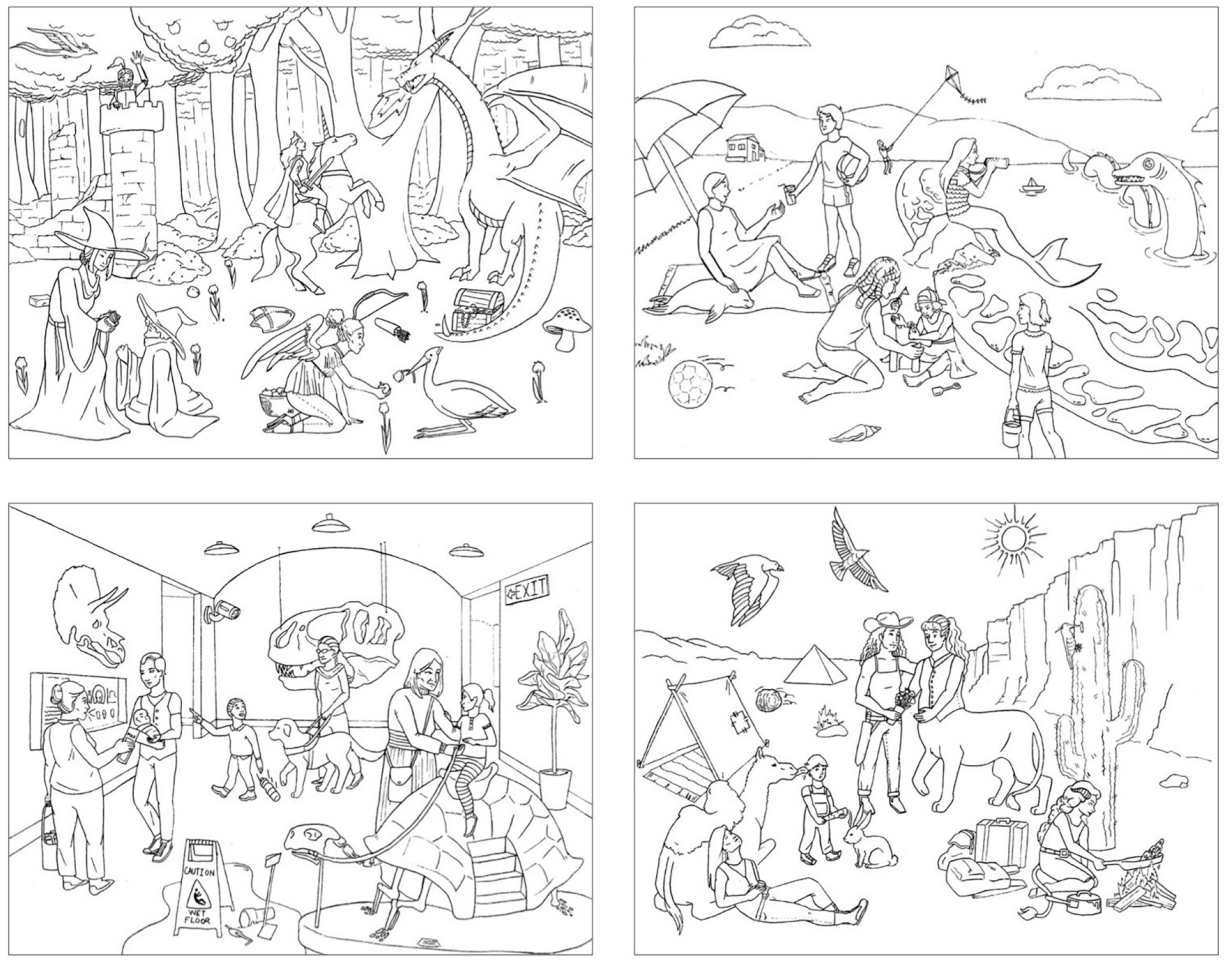
Experimental images for the picture description task.

The picture description stimuli comprised one practice image and four experimental images. The experimental images were created to emulate the classic “Cookie Theft” image from the Boston Diagnostic Aphasia Examination (Goodglass & Kaplan, 1972) in that each image depicts some overarching scene with many supporting details. In an adaptation of the criteria used by Catricalà and colleagues (2017), each of the experimental images depicted the following: 8 subjects (all animate nouns), 10 actions (at least 2 transitive and 2 ditransitive), and 13 unique objects (Figure 1).

#### Cognitive control tasks

For the AX-CPT, each trial consisted of a series of five letters: a red cue letter, followed by three black distractor letters and a red probe letter. There were four trial types: target AX trials (where A appeared as the cue and X appeared as the probe) and non-target AY trials (where A appeared as the cue and a non-X letter of the alphabet appeared as the probe), BX trials (where a non-A letter of the alphabet appeared as the cue and X appeared as the probe), and BY trials (where neither the cue nor the probe were A or X). The letter A never appeared in the probe position, and the letter X never appeared in the cue position. Furthermore, the letters K and Y never appeared during the letter sequence due to their perceptual similarity to the letter X.

The Flanker task consisted of congruent and incongruent trials. Each trial contained a row of five angle brackets. For the congruent trials, the centre bracket and the four flanking brackets faced the same direction (e.g., <<<<<). For the incongruent trials, the centre bracket and the four flanking brackets faced opposite directions (e.g.,<<><<). Thus, the incongruent trials required suppression of the flanking brackets.

The counting Stroop task consisted of neutral and incongruent trials. For the neutral trials, the words on the screen were all monosyllabic animal words (e.g., *cat cat*). For the incongruent trials, the words on the screen were monosyllabic number words (e.g., *one one one*). Thus, incongruent trails required the suppression of the semantic content of the number words.

### Procedure

Procedures were approved by Penn State’s institutional review board (IRB). After providing verbal consent, participants completed the picture description task. We utilised a picture description task over other common speech elicitation techniques (e.g., expository discourse tasks) because it allows for more control over the semantic content of participants’ productions. Controlling the semantic content allowed for a greater focus on the syntax of participants’ productions. The picture description task was administered over Zoom with the experimenter present. Participants were explicitly asked to wear headphones for the picture description task to ensure that the background noise would not interfere with the audio recordings. During this task, participants orally described the set of images in as much detail as possible. The images were described against (relative) silence or noise, alternating the two conditions for each image. During the noise condition, 16-talker babble (Harris, 2018) consisting of 8 male talkers and 8 female talkers was played over participants’ headphones. This babble was unintelligible and devoid of syntactic and semantic information but maintained speech-like acoustic properties, making it more cognitively demanding to listen to than other types of noise (e.g., white noise; Danhauer & Leppler, 1979). Both the order of the experimental images and whether they were paired with noise or silence were counterbalanced across participants.

Before starting the picture description task, the experimenter worked with the participant to test the loudness of the background noise. The experimenter played the background noise audio file on their computer and shared the background noise with the participant through Zoom. While wearing headphones, participants were asked to adjust the volume of their own computer until they felt that the loudness of the noise had reached a “loud party level,” meaning that the noise was loud enough to be distracting and difficult, but that it was still possible to converse. Crucially, the experimenter made it clear to the participant that they were also able to hear the background noise by increasing vocal intensity while speaking to the participant during the background noise adjustment process. Considering that speakers could alter the complexity of their speech to ease their listener’s comprehension, we wanted to ensure that participants were aware that the experimenter could hear the background noise. Although volume control was admittedly imprecise, one benefit of our method was that noise levels were tailored to each individual’s perceived threshold of distractibility. Once the volume level was adjusted, participants were explicitly told not to change the volume of their computer for the duration of the study.

Participants were instructed to describe each image for at least 2 min 30 s, but no more than 5 min. A small timer was present in the lower right-hand corner of the screen so that participants could monitor how long they had been describing each image. Of the 57 participants, 1 participant did not meet the minimum speech duration requirement for one of the images, speaking for only 1 min and 30 s. In addition, one participant exceeded the maximum speech duration requirement when describing one of the images, speaking for 5 min 30 s. All results presented below include data from these two individuals who did not comply with the minimum and maximum speech duration requirements, but we also ran all complexity analyses with these two individuals excluded. With the exception of the T-unit and average unfilled pause duration complexity analyses, in which the main effect of Condition became non-significant when excluding these two participants, the same pattern of results reported below holds.

During the picture description task, participants were informed that the experimenter would be taking notes by hand as the participant described each image. This procedure was intended to encourage the participant to consider their addressee’s (i.e., the experimenter’s) communicative needs during the task. Furthermore, and in contrast to related prior work (Harmon et al., 2021; Kemper et al., 2003), participants were made aware that the experimenter could hear the background noise. After providing instructions to the participant, both the experimenter and the participant turned off their cameras so that participants could not rely on facial cues as a means of mitigating noise-induced communication difficulties. The experimenter (i.e., the listener) remained silent as the participant described each image. Participants’ speech was recorded using the audio record function in Zoom.

**Figure 2.**
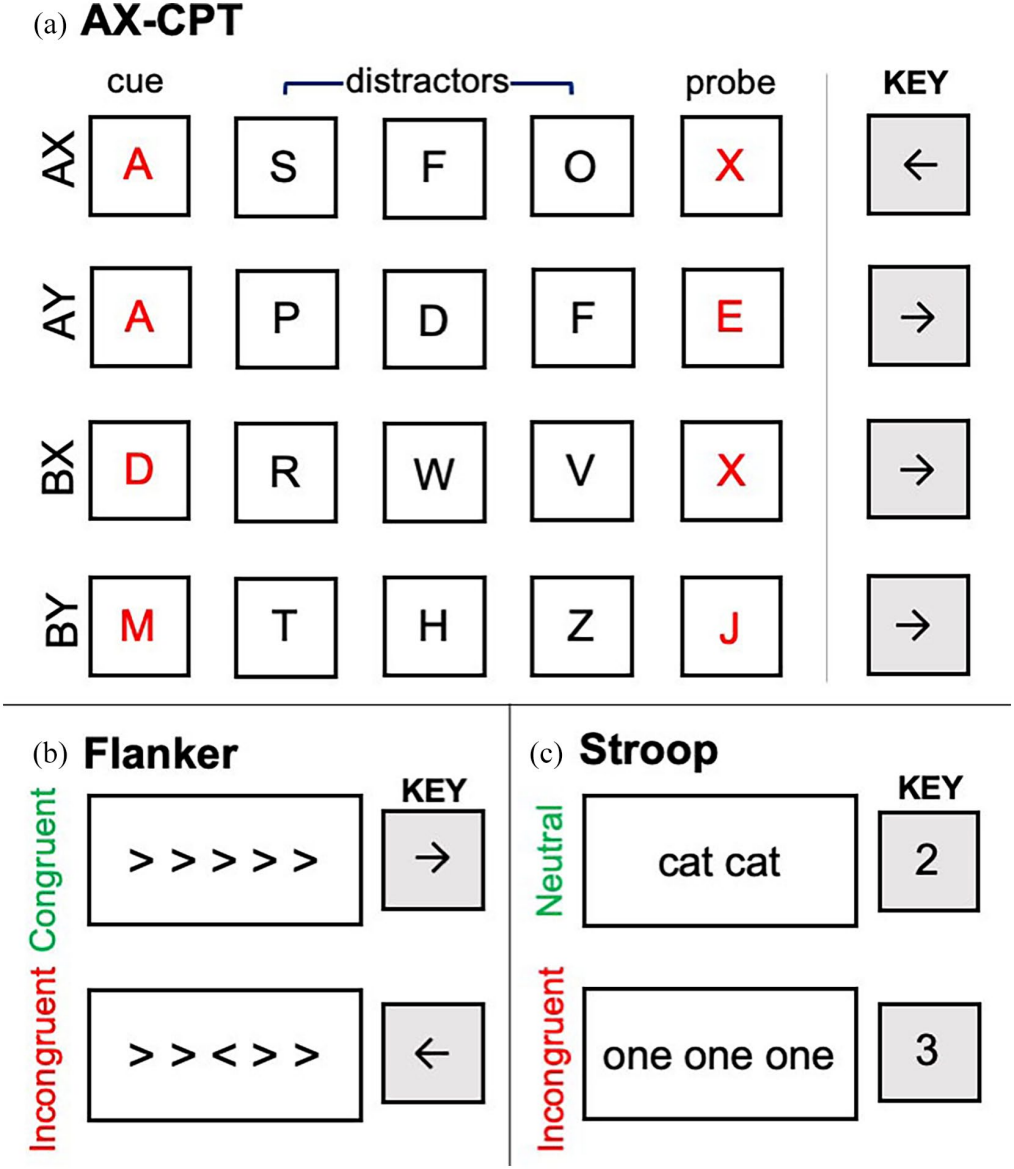
Visualisations of each trial types in each cognitive control task. During the AX-CPT, participants pressed the right arrow key in response to all cue and distractor letters. Crucially, in response to the probe letter, participants only pressed the left arrow key if the red cue–probe pair corresponded to an AX pair, as shown in (a). During the Flanker task, participants responded to the direction of the middle arrow as shown in (b). During the Stroop task, participants responded to the number of words on the screen, as shown in (c).

Following the picture description task, participants completed a battery of cognitive control tasks: the AX-CPT, Flanker task, and the counting Stroop task. The cognitive control tasks were administered online via Pavlovia, an online platform for hosting experiments built in PsychoPy (Peirce et al., 2019). The order of the cognitive control tasks was counterbalanced across participants. Following the protocol outlined in Karuza et al. (2016), the Flanker task consisted of an equal number (*n* = 188) of congruent and incongruent trials. Participants were instructed to press either the right or left arrow keys to indicate which direction the centre bracket was facing (Figure 2a). Each trial was displayed for 800 ms followed by a fixation cross with an ISI interval drawn from a uniform distribution (500–1,250 ms). Congruent and incongruent trials were randomly interleaved, and reaction times and accuracy were recorded.

The counting Stroop task (Bush et al., 2006) consisted of 8 blocks of 20 trials with alternating neutral and incongruent blocks. Participants were instructed to count the total number of words presented on the screen, ranging from 1 to 4, and push the corresponding number key on their keyboard (Figure 2b). Each trial was displayed for 1,500 ms with an ISI interval drawn from a uniform distribution (500–1,250 ms). Reaction times and accuracy were recorded.

The AX-CPT (Braver et al., 2001) consisted of 80 trials. Each trial consisted of a series of five letters presented one at a time on the centre of the screen: a red cue letter, followed by three black distractor letters, and a red probe letter. Following all cue and distractor letters, participants were instructed to press the right arrow key. Following the probe letter, participants were asked to change their response and press the left arrow key if the probe was an X that was preceded by an A cue (Figure 2c). Otherwise, participants were instructed to press the right arrow key; 70% of all trials consisted of AX trials (*n* = 56), and 30% consisted of an equal number of trials (*n* = 8) for the remaining three trial types (AY, BX, and BY). Reaction times and accuracy were recorded.

### Transcription of speech production data

Production data were transcribed verbatim by the first author (C.T.P.) or a research assistant. Because it is notoriously difficult to identify sentence boundaries in spoken language, the transcriptions were segmented into minimal terminable units (T-units), which consist of a main clause and any subordinate clauses or non-clausal structures attached to it (Hunt, 1970). For utterances that contained coordinate main clauses, if a subject was explicitly produced in the first main clause but not in the second clause the utterance was considered one T-unit (e.g., *the woman is petting a seal and is holding a drink in her hand*). However, if a subject was explicitly produced in both clauses, the utterance was segmented into two T-units (e.g., *the woman is petting a seal* / *and she is holding a drink in her hand*). Utterances that lacked an explicit subject and/or a verb were considered fragments and placed in parentheses and excluded from analysis. However, if the subject of the utterance could be inferred based on previous context, that utterance was considered a T-unit (e.g., *the older woman could be her mother or grandmother / [she] has shoulder_length hair*). Clauses were coded in accordance to the guidelines from Nippold and colleagues (2014), identifying main, adverbial, relative, nominal, infinitive, participial, and gerund clauses. Pairs of words were treated as compound words if they were included as lexical entries in Merriam-Webster’s Dictionary, in which case these words would be linked using an underscore (e.g., *beach ball* transcribed as *beach_ball*). While an imperfect method (e.g., *beach ball* is listed as a lexical entry but *soccer ball* is not), this approach ensured that semantically linked word pairs would be transcribed consistently across participants. Proper nouns were also treated as one word and linked with an underscore (e.g., *Loch Ness Monster* transcribed as *Loch_Ness_Monster*). For the purposes of our analyses, filled and unfilled pauses and linguistic mazes were treated as disfluencies and placed in parentheses. Linguistic mazes refer to words or sentence fragments that disrupt the flow of speech and do not contribute to the speaker’s intended message (see Loban, 1976). Researchers slightly vary in how they define and categorise mazes (Bangert & Finestack, 2020), but for our purposes, we use the term *maze* to refer to abandoned words/utterances, false starts/reformulations, and repetitions (see discussion of mazes under the “Measures of Disfluencies and Errors” section). Grammatical errors were annotated (see discussion of errors under the “Measures of Disfluencies and Errors” section). All transcription files were double checked by at least one other member of the research team.

### Measures of complexity

#### Ratio-based measures

MLTU was calculated by dividing the total number of words by the total number T-units in a language sample. CD was calculated by dividing the total number of clauses (main and subordinate) by the total number of T-units (Hunt, 1965). Disfluencies (filled pauses and mazes) were removed before calculating MLTU and CD.

#### Measures of quantity

We computed average number of T-units, clauses, and words produced by participants in each condition, after the removal of all filled pauses and mazes.

#### Measures of disfluencies and errors

We divided the category of disfluencies into two subcategories: pauses and mazes. For our analyses, we make the distinction between unfilled (silent) pauses and filled pauses (*um, uh, hm, ah, er*) because the two been argued serve different functions in speech production. Under some accounts, unfilled pauses are considered a proxy for a speaker’s difficulty in utterance planning (e.g., Goldman-Eisler, 1968), whereas filled pauses are thought to be produced for the listener’s benefit (e.g., Clark & Fox Tree, 2002). Thus, we calculated the average number of unfilled and filled pauses separately. In addition, we also calculated average unfilled pause duration to account for the possibility that participants may differ in how long they silently pause in each condition. In accordance with Goldman-Eisler’s (1968) standard 250 ms threshold for distinguishing between pauses associated with articulation (<250 ms) and hesitations (⩾250 ms) in speech production, our calculations for number of unfilled pauses and duration of unfilled pauses only included pauses greater than or equal to 250 ms in length. In addition to filled and unfilled pauses, our other measure of disfluency was number of mazes, which encompasses abandoned words/utterances (e.g., ***I wonder-*** or ***banan-***), false starts/reformulations (the speaker revises their utterance; e.g., ***he is*** *he dropped his water bottle*), repetitions (the speaker reproduces the word/phrase verbatim; e.g., ***there’s a*** *there’s a mermaid on a rock*). Adjacent mazes were combined, so if multiple mazes appeared in row, they were placed within the same set of parentheses and only counted once.

We also measured the number of errors participants produced. Although participants did produce some phonetic and semantic errors during the picture description task, we focused on syntactic errors in the present analyses. Syntactic errors included incorrect subject-verb agreement (e.g., *there* ***is hills*** *in the background*), the production of an unnecessary contraction (e.g., *she has a purse with her* ***that’s looks*** *very full*), the insertion of an unnecessary word that renders the T-unit ungrammatical (e.g., *it looks like mountains in the background which makes me* ***to*** *believe that it’s a lake*), or the omission of a single word (e.g., *I’m gonna assume it’s* ***[a]*** *pretty windy day*). Although we initially did not intend to conduct a separate analysis of number of omissions, we opted to do so after seeing the prevalence of both function word omissions (e.g., *I’m gonna assume it’s* ***[a]*** *pretty windy day*) and subject omissions (e.g., *the older woman could be her mother or grandmother /****[she]*** *has shoulder_length hair*) in the data).

## Results

### The effect of background noise on speech production

#### Acoustic analyses

Given that data were collected remotely through Zoom, one potential concern is the lack of control the experimenter had over the participants’ noise levels. Thus, because it is well attested that speakers exhibit the Lombard effect when speaking in noisy environments (e.g., Castellanos et al., 1996; Junqua, 1993; Lombard, 1911; Pisoni et al., 1985; Summers et al., 1988), we examined whether our participants exhibited evidence of speaking louder during the noise condition relative to silence condition. Generally, greater intensity in the noise relative to silence condition would serve as strong evidence that participants were complying with task instructions. We used Praat (v. 6.1.41; Boersma & Weenink, 2021) to calculate mean intensity and peak intensity of participants’ speech production. Because participants varied in the number and duration of unfilled (silent) pauses that they produced, we calculated mean intensity with unfilled pauses included as well as with unfilled pauses with a duration of greater than 250 ms excluded. Overall, participants exhibited higher mean intensities in the noise condition (without unfilled pauses: *M* = 70.99 dB, *SD* = 1.82 dB; with unfilled pauses: *M* = 69.76 dB, *SD* = 1.77 dB) than in the silence condition (without unfilled pauses: *M* = 69.85 dB, *SD* = 1.81 dB; with unfilled pauses: *M* = 68.63 dB, *SD* = 1.79 dB). Mean intensities were significantly different between the two conditions (without pauses: *M*_difference_ = 1.13 dB, *SD* = 1.31 dB, 95% CI [0.79, 1.48], *t*(56) = 6.53, *p* < .0001; with pauses: *M*_difference_ = 1.13 dB, *SD* = 1.35 dB, 95% CI [0.77, 1.49], *t*(56) = 6.33, *p* < .0001). When normality was assessed using the Shapiro–Wilk’s test, peak intensity deviated from normal, so a non-parametric test was applied for the noise versus silence comparison for peak intensity. The Wilcoxon signed-rank test indicated that peak intensity also significantly differed between the two conditions (*M*_difference_ = 0.55 dB, *SD* = 1.13 dB, 95% CI [0.84, 0.24], *p* = .003) such that participants exhibited a significantly higher peak intensity in noise (*M* = 82.15 dB, *SD* = 1.23 dB) compared with silence (*M* = 81.60 dB, *SD* = 1.56 dB). In sum, when considering all three types of intensity measurements (mean intensity with unfilled pauses removed, mean intensity with unfilled pauses included, and peak intensity), participants generally spoke louder in the noise condition compared with silence.

Because the effect of noise on speech production varies across speakers (Junqua, 1993), we differentiated between participants who were more strongly affected by the background noise (at least at the acoustic level) and those who were not . We calculated the difference in mean intensity (with unfilled pauses removed) between the noise and silence condition for each participant. In all, 47 participants had positive mean intensity differences (indicating that they spoke louder in the noise condition) and 10 participants had negative mean intensity differences (indicating they spoke louder in the silence condition). Although the results presented below include all 57 participants, we also ran the analyses reported below with the subset of 47 participants who spoke louder in the noise condition, and, with the exception of T-unit and average unfilled pause duration analyses, in which the main effect of Condition became non-significant after excluding these participants, the same pattern of results hold.

#### Complexity analyses

In the present study, our primary comparison of interest was the difference between speech produced in the noise relative to the silence condition. For each of our 11 complexity metrics, we implemented a linear mixed effects model (LMM) using the *lmer()* function in the lme4 package (v. 1.1-26; Bates et al., 2015) in R (v. 4.0.4; R Core Team, 2021). Statistical significance was evaluated using the R lmerTest package (v. 3.1-3; Kuznetsova et al., 2017). Each complexity metric was regressed onto all main effects and interactions of Condition (noise vs silence) and Run (1–4), which was included to account for the possibility that participants’ performance during the picture description task might improve as they described more images. Each model included the fullest justified random effects structure that allowed the model to converge. Details of our method of model building are provided in Section 1 of the Supplemental Materials. For Model 8 (Filled Pauses) and Model 9 (Mazes), the fullest random effects structure corresponded to a random intercept for Participant. Models 10 (Errors) and 11 (Omissions) included random intercepts for Participant and Picture (item) and a by-Participant random slope for Condition (Model 1—MLTU—instead included a by-Participant random slope for Run). Models 2 (CD), 3 (T-units), 5 (Words), and 6 (Unfilled Pauses) included random intercepts for Participant and Picture and by-Participant random slopes for Condition and Run. Finally, for Models 4 (Clauses) and 7 (Average Unfilled Pause Duration) the fullest random effects structure corresponded to random intercepts for Participant and Picture, by-Participant random slopes for Condition and Run, and a by-Picture random slope for Run.

**Figure 3.**
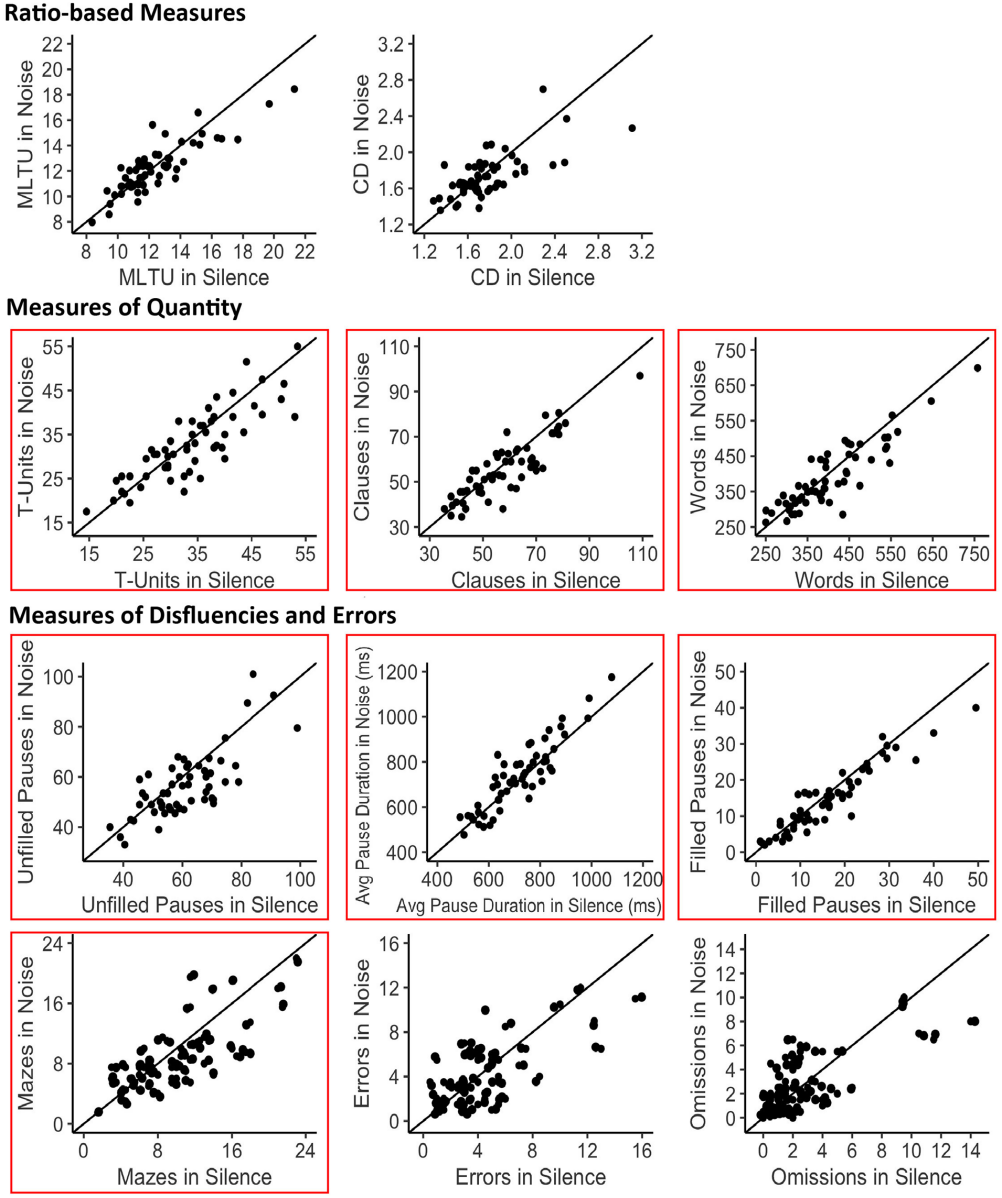
Two-dimensional scatterplots depicting differences between the noise and silence conditions for each complexity measure. Each point represents one participant. The diagonal line represents a zero difference between the noise and silence condition. The greater the distance between a point and the diagonal line, the greater the difference between the noise and silence condition for that individual participant. The boxes signify complexity measures that exhibited a significant main effect of Condition (*p* < .05).

Because we were principally interested in the main effect of Condition on our complexity measures, we summarise these results in Table 1. For the full summary of the analyses, see Table S1 in the Supplemental Materials. Contrary to our original prediction, a significant main effect of Condition was not observed for our ratio-based measures of complexity. Participants did not produce longer T-units (MLTU: β = .10, *t* = 0.99, *p* = .32), nor did they produce more clauses per T-unit in silence compared with noise (CD: β = 0.02, *t* = 1.53, *p* = .13). For measures that captured the overall quantity of speech produced, we observed a significant main effect of Condition for number of T-units (β = 0.70, *t* = 2.19, *p* = .03), number of clauses (β = 1.49, *t* = 3.22, *p* = .002), and number of words (β = 9.53, *t* = 2.98, *p* = .004). Notably, participants produced fewer T-units, clauses, and words in noise compared with silence (Figure 3).

**Table 1.** Coefficients and corresponding *t* values and *p* values for the main effect of Condition in a linear mixed effects model examining the effect of Condition and Run on 11 measures of complexity.

Complexity measure	Coefficient	*t* value	*p* value
Ratio-based measures			
MODEL 1: *Mean Length of T-unit*	0.10	0.99	.32
MODEL 2: *Clausal Density*	0.02	1.53	.13
Measures of quantity			
**MODEL 3: *Number of T-units***	**0.70**	**2.19**	**.03**
**MODEL 4: *Number of Clauses***	**1.49**	**3.22**	**.002**
**MODEL 5: *Number of Words***	**9.53**	**2.98**	**.04**
Measures of disfluencies and errors			
**MODEL 6: *Number of Unfilled Pauses***	**2.60**	**4.33**	**<.0001**
**MODEL 7: *Average Unfilled Pause Duration***	**–12.08**	**–2.27**	**.03**
**MODEL 8: *Number of Filled Pauses***	**0.79**	**3.35**	**<.001**
**MODEL 9: *Number of Mazes***	**0.62**	**2.50**	**.01**
MODEL 10: *Number of Errors*	0.03	0.17	.87
MODEL 11: *Number of Omitted Words*	0.03	0.20	.84

Significant values are bolded. To see the full summary of the results, including the main effect of Run and the interaction between Condition and Run, refer to Table S1 in the Supplemental Materials.

For the disfluency measures, we observed a significant main effect of Condition for number of filled pauses (β = 0.79, *t* = 3.35, *p* < .001), unfilled pauses (β = 2.60, *t* = 4.33, *p* < .0001), and mazes (β = 0.62, *t* = 2.50, *p* = .01), indicating that participants produced fewer filled pauses, unfilled pauses, and mazes when speaking in noise compared with silence (Figure 3). Crucially, the significant effect of Condition for number of filled pauses, unfilled pauses, and mazes was maintained even after accounting for number of T-units, clauses and words in the models (filled pauses: β = 0.69, *t* = 2.94, *p* = .004; unfilled pauses: β = 1.90, *t* = 3.78, *p* < .001; mazes: β = 0.51, *t* = 2.02, *p* = .045). In other words, participants’ tendency to produce fewer filled pauses, unfilled pauses, and mazes in noise cannot solely be attributed to the fact that they also produced fewer clauses and words in noise. The effect of Condition on average unfilled pause duration was significant (β = –12.08, *t* = –2.27, *p* = .03), indicating that, although participants paused less frequently in noise, individual pauses were longer. Participants did not exhibit a significant difference in the number of errors (β = 0.03, *t* = 0.17, *p* = .87) nor the number of omissions (β = 0.03, *t* = 0.20, *p* = .84) that they produced in noise relative to silence.^1^

### The role of cognitive control in speech production in noise

Because we were also interested in examining the effect of cognitive control on speech production in noise versus silence, we computed correlations between the cognitive control measures and the complexity measures that significantly differed between the noise and silence conditions (T-units, Clauses, Words, Unfilled Pauses, Average Unfilled Pause Duration, Filled Pauses, and Mazes).

#### AX-CPT

Prior to analysis of the AX-CPT data, to eliminate participants who did not comply with task instructions, we excluded participants who did not meet an overall accuracy threshold of 70%. This approach led to the exclusion of five participants (*N* = 52). After the exclusion of these participants, overall performance on the AX-CPT was relatively high (mean accuracy = 90.12%, *SE* = 0.83). For each remaining participant, we then also excluded any RTs less than 200 ms (5.79% data loss) and greater than 1,300 ms (1.59% data loss; based on the trial timing of Braver et al., 2001). Analyses were conducted utilising RTs only for correct trials.

During the AX-CPT, participants may have adopted different control strategies while completing the task. Some participants may have relied on proactive goal maintenance, preparing key responses to the probe after encountering the cue. Other participants may have relied on reactive inhibitory control, not preparing key responses to the probe after encountering the cue, resulting in longer RTs and more errors for BX trials. To quantify individual participants’ bias towards one control strategy over the other, we calculated the Behavioral Shift Index (BSI; Braver et al., 2009) for RTs using the following formula (AY – BX)/(BX + BY). Using *z*-score transformed RTs and percent error, we calculated a composite BSI score. A positive BSI score indicates reliance on proactive control, whereas a negative BSI score indicates reliance on reactive control.

**Figure 4.**
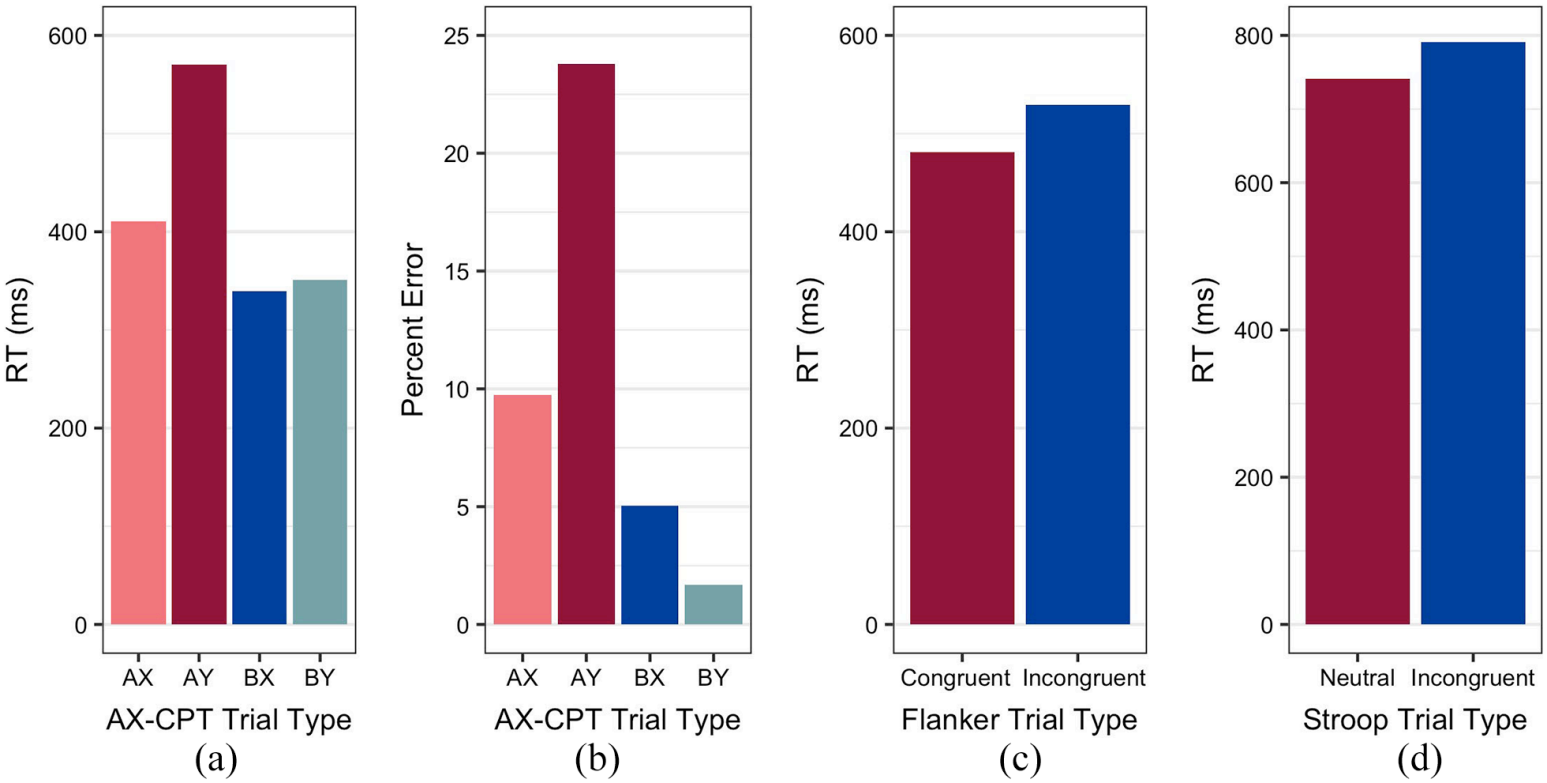
Bar graphs comparing (a) RTs and (b) percent error for each trial type in the AX-CPT, (c) RTs for the Flanker task, and (d) RTs for the Stroop task. As these represent within-participant measures, error bars are not included.

Focusing on comparisons between our two principal trial types of interest, we found that participants had longer RTs on AY trials (*M* = 570.23 ms, *SD* = 138.30 ms) than on BX trials (*M* = 339.70 ms, *SD* = 86.76 ms). RTs differed significantly between these two trial types (*M_difference_* = 230.53 ms, *SD* = 133.43 ms, 95% CI [193.38, 267.68], *t*(51) = 12.46, *p* < .0001); see Figure 4a. We assessed accuracy by implementing a generalised linear mixed effects model (GLMM) using the g*lmer()* function in the lme4 package (v. 1.1-26; Bates et al., 2015). Accuracy was regressed onto all main effects and interactions of Trial Type (AY versus BX) and Trial Number (1–80), which was included to account for the possibility that participants’ performance might improve as they completed more trials. The fullest random effects structure corresponded to a random intercept for Participant. We observed a significant main effect of Trial Type (β = 0.93, *z* = 6.82, *p* < .0001), indicating that participants exhibited a higher percentage of errors (i.e., lower accuracy) for AY trials (*M* = 23.80%, *SD* = 18.57%) than BX trials (*M* = 5.05%, *SD* = 7.11%) (Figure 4b). An additional main effect of Trial Number was observed (β = –0.02, *z* = −2.68, *p* = .007), indicating that accuracy generally decreased as participants completed more trials.

For each of the complexity measures that exhibited a significant main effect of Condition (T-units, Clauses, Words, Unfilled Pauses, Average Unfilled Pause Duration, Filled Pauses, and Mazes), we examined whether the magnitude of the difference between the noise and silence conditions was correlated with BSI. We did not find evidence of a correlation between these measures and BSI scores (see Table S2 in the Supplemental Materials). Neither a reliance on proactive control nor a reliance on reactive control significantly correlated with participants’ tendency to produce fewer T-units, clauses, words, unfilled pauses, filled pauses, and mazes or the tendency to produce longer unfilled pauses in noise compared with silence.

#### Flanker task

Prior to analysis of the Flanker data, to eliminate participants who did not comply with task instructions, we excluded participants who did not meet an overall accuracy threshold of 70%. This approach led to the exclusion of 13 participants (total remaining participants for analysis, *N* = 44). After the exclusion of these participants, overall performance on the Flanker was quite high (mean accuracy = 90.12%, *SE* = 0.68). For each remaining participant, we then also excluded RTs less than 200 ms (0.10% data loss). Very long RTs were not excluded because trial durations were capped at 800 ms for the Flanker task (Karuza et al., 2016). If participants did not respond within this time limit, the trial was scored as incorrect. Analyses were conducted utilising RTs only for correct trials.

Given an observed deviation from normality, a non-parametric test was used to compare the incongruent versus congruent trial types. As expected, participants exhibited longer RTs for incongruent trials (*M* = 529.42 ms, *SD* = 36.99 ms) compared with congruent trials (*M* = 481.02 ms, *SD* = 34.15 ms) (Figure 4c). RTs differed significantly between the two trial types, as assessed by the Wilcoxon signed-rank test (*M*_difference_ = 48.40 ms, *SD* = 21.97 ms, 95% CI [41.72, 55.07], *p* < .0001). For each of the complexity measures that exhibited a significant main effect of Condition (T-units, Clauses, Words, Unfilled Pauses, Average Unfilled Pause Duration, Filled Pauses, and Mazes), we examined whether the magnitude of the difference between the noise and silence conditions was correlated with the RT interference effect (obtained by calculating the difference in RTs between incongruent and congruent trials). No significant correlation was observed between each of these measures and RT interference (Table S2, Supplemental Materials).

#### Number Stroop task

All participants achieved an overall accuracy score of at least 70%, so we did not exclude any participants from the Stroop analyses (*N* = 57). Overall performance on the Stroop task was quite high (mean accuracy = 92.87%, *SE* = 0.55). For each remaining participant, we then also excluded RTs less than 200 ms (0.08% data loss). Very long RTs were not excluded because trial durations were capped at 1500 ms for the Stroop task (Bush et al., 2006). If participants did not respond within this time limit, the trial was scored as incorrect. Analyses were conducted utilising RTs only for correct trials.

**Figure 5.**
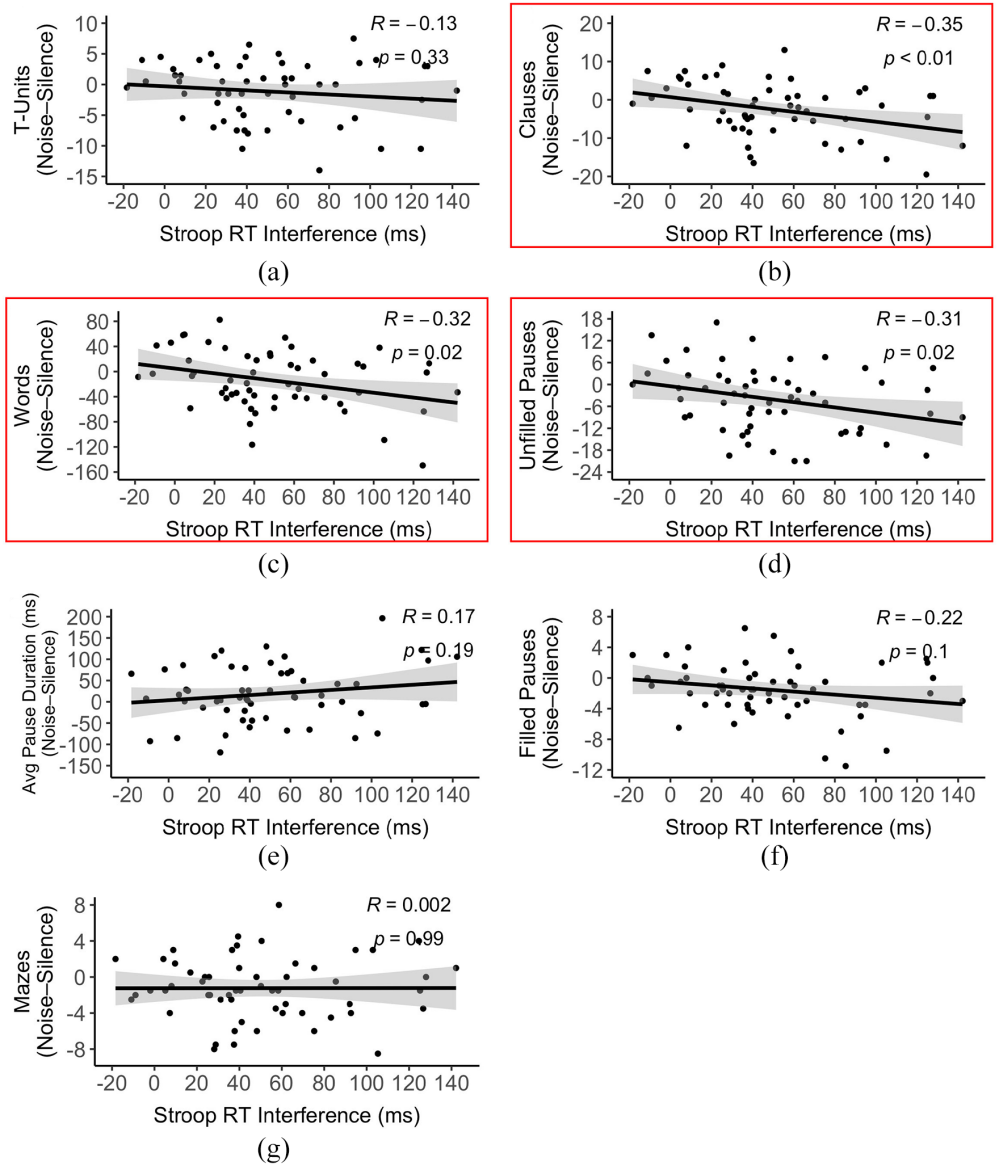
Scatterplots depicting correlations between Stroop RT interference and noise-induced differences in number of (a) T-units, (b) clauses, (c) words, (d) unfilled pauses, (e) average unfilled pause duration, (f) filled pauses, and (g) mazes. The boxes signify complexity measures that exhibited a significant correlation with Stroop RT interference (*p* < .05).

As expected, participants exhibited longer RTs on incongruent trials (*M* = 791.14 ms, *SD* = 85.55 ms) than on neutral trials (*M* = 741.11 ms, *SD* = 88.26 ms). RTs significantly differed between these two trial types (*M*_difference_ = 50.04 ms, *SD* = 37.98 ms, 95% CI [39.96, 60.11], *t*(56) = 9.95, *p* < .0001). For each of the complexity measures that exhibited a significant main effect of Condition (T-units, Clauses, Words, Unfilled Pauses, Average Unfilled Pause Duration, Filled Pauses, and Mazes), we examined whether the magnitude of difference between the noise and silence conditions was correlated with the RT interference effect (obtained by calculating the difference in RTs between incongruent and neutral trials) on the Stroop task. Stroop RT interference was not significantly correlated with differences in number of T-units, (*r* = −.13, *p* = .33), average unfilled pause duration (*r* = .17, *p* = .19), number of filled pauses (*r* = −.22, *p* = .10), or mazes (*r* = .002, *p* = .99), but it was significantly correlated with differences in number of clauses (*r* = −.35, *p* = .008), words (*r* = −.32, *p* = .02), and unfilled pauses (*r* = −.31, *p* = .02). Individuals who exhibited larger interference effects, suggestive of weaker cognitive control, produced fewer clauses, words, and unfilled pauses when speaking in noise than silence (Figure 5). We note that the significance of these correlations does not hold when subjected to a conservative multiple comparisons correction (i.e., the Bonferroni correction).

While there is precedent for quantifying individual differences in cognitive control through RT interference effects (e.g., Capizzi et al., 2017; Kiefer et al., 2005; Rodriguez-Fornells et al., 2012; Vuong & Martin, 2014), Draheim and colleagues (2019) have raised important concerns about the reliability of difference scores when used in correlational analyses focused on individual differences. To supplement the simple correlation analyses illustrated in Figure 5—in which by-participant mean differences in complexity for noise versus silence served as the dependent measure—we added Stroop RT Interference as a predictor into the seven LMMs for which we had observed a significant main effect of Condition (Table 1). A significant interaction between Condition and Stroop Interference was not observed for number of T-units (β = 0.01, *t* = 1.68, *p* = .10), filled pauses (β = 0.01, *t* = 1.63, *p* = .10), or mazes (β = −0.00002, *t* = −0.004, *p* = .997). A marginal interaction between Condition and Stroop Interference was observed for average unfilled pause duration (β = −0.27, *t* = −1.86, *p* = .07). As expected, we did observe a significant interaction between Condition and Stroop Interference for number of clauses (β = 0.04, *t* = 2.94, *p* = .004), number of words (β = 0.20, *t* = 2.51, *p* = .02), and number of unfilled pauses (β = 0.04, *t* = 2.86, *p* = .006). Subsequent simple effects analyses revealed the Stroop Interference effect was not specific to a single level of Condition considered alone (e.g., noise or silence); however, the effect of Interference on number of unfilled pauses in noise was marginal (β = −0.09, *t* = −1.98, *p* = .05).

Under this approach, we were able to account for participant- and item-level random effects without relying on mean difference scores for the complexity metrics (Rouder & Haaf, 2019). However, this approach continued to rely on difference scores for the Stroop RT interference predictor. Although lack of a correlation between linguistic and non-linguistic cognitive control tasks is commonly reported (e.g., Fan et al., 2003; Kousaie & Phillips, 2012; Mendoza et al., 2021), concerns about the reliability of these Stroop difference scores are potentially compounded by the observation that performance on the Stroop task was neither significantly correlated with BSI scores on the AX-CPT (*r* = −.20, *p* = .20) nor with the RT interference effect measured via the Flanker task (*r* = −.14, *p* = .39). Stroop RT difference scores were assessed through permutation-based split-half reliability (Parsons et al., 2019, see also Pronk et al., 2022) using the *splithalf()* function in the splithalf package (v. 0.7.2; Parsons, 2021) in R. Reliability was averaged across 5000 random splits, and then adjusted using the Spearman-Brown prophecy formula to reflect the full length of the test. Stroop RTs for individual trial types were reliable (congruent: coefficient = 0. 96, 95% CI [0.94, 0.97]; incongruent: coefficient = 0. 94, 95% CI [0.91, 0.96]). RT difference scores were less reliable (coefficient = 0.46, 95% CI [0.23, 0.65]). Strauss et al. (2005) similarly reported a test–retest reliability coefficient of .46 for colour-word Stroop RT difference scores, which they considered an indication of moderate reliability. However, we acknowledge that reliability of Stroop RT difference scores falls below the generally accepted reliability threshold of .7 to .8, a caveat to bear in mind when interpreting the individual differences-based correlational analyses presented here. In Section 4 of the Supplemental Materials, we expand on this issue and explore alternative methods of assessing individual differences in Stroop performance.

### Post hoc analyses: accounting for quantity differences between noise and silence

#### Hedges and lexical fillers

The results reveal that participants clearly produced less in noise, but the driving force behind this change remains unclear. Were these adjustments for their own benefit, due to the distracting nature of noise, or were they streamlining their speech, potentially, for the listener’s benefit? To further probe why speakers reduced the number of words and clauses produced in noise, we conducted a follow-up analysis to examine whether the number of hedges (e.g., *I think; it seems; it looks likes*) differed between noise and silence conditions. The use of words or phrases to convey uncertainty, possibility, and probability is referred to as hedging, and this behaviour has been argued to be characteristic of women’s speech (e.g., Holmes, 1990; Mondorf, 2002)—important consideration given that 90% of our participants were female. From the listener’s perspective, knowledge of the speaker’s uncertainty, while informative, is not essential for comprehending the speaker’s picture descriptions. A reduction in hedges would reduce the amount of information the listener needs to process in noise and might be driving noise-induced reductions in words and clauses. Hedges have been divided into relational hedges and propositional hedges (Prokoﬁeva & Hirschberg, 2014; Ulinski & Hirschberg, 2019). Relational hedges introduce the speaker’s own uncertainty in relation to the propositional content of their utterance (e.g., *it* ***looks like*** *it’s a windy day*) whereas propositional hedges introduce uncertainty into the propositional content itself (e.g., *it’s* ***kind of*** *a windy day*). We opted to focus on relational hedges in part due to their prevalence in the production data (Section 2, Supplemental Materials), but also because, for the listener, knowledge of the speaker’s uncertainty, while informative, is not essential for comprehending the speaker’s description of the images. If speakers were streamlining their speech in noise to benefit the listener, we would expect speakers to produce fewer hedges in noise. Although results from the LMM did reveal participants produced fewer hedges in noise than in silence (β = 0.41, *t* = 1.83, *p* = .07), this effect was marginally significant.

We also examined whether speaking condition affected the production of lexical fillers because they were generally included when calculating number of words produced. We included words/phrases that are commonly considered lexical fillers in our analysis (*alright, I mean, like, okay, so, well*, and *you know*) (Bolden, 2009; Filipi & Wales, 2003; Fuller, 2003; Laserna et al., 2014). Note that *alright* and *okay* almost exclusively produced at the beginning of a transcription before participants began describing the image and were therefore placed in parentheses and excluded from total word counts. Nevertheless, we included them in our analysis of lexical fillers. Results from the LMM revealed participants did not differ in their production of lexical fillers between the noise and silence conditions (β = 0.30, *t* = 1.65, *p* = .10). Taken together, the results of these follow-up analyses suggest that the decreased number of words produced in noise is not strongly tied to a decrease in number of hedges or lexical fillers in noise.

#### Articulation rate

Another potential explanation for the observed decrease in words and clauses produced in noise may be that speakers were speaking more slowly during the noise condition. Given that participants were asked to speak for a maximum of 5 min, speaking more slowly in noise could account for quantity differences in the amount of speech produced between noise and silence. Alternatively, speaking more quickly may be another means of maintaining focus on the task in the face of distraction. Thus, we conducted additional follow-up analyses on articulation rate to explore these two possibilities. Articulation rate was calculated by dividing the total number of words by the total phonation time (i.e., duration of the recording with unfilled pauses removed). Overall, participants spoke less during the noise condition (*M* = 2.13 min, *SD* = 0.40 min) than the silence condition (*M* = 2.23 min, *SD* = 0.49 min). The difference in phonation time between the noise and silence conditions was significant (*M*_difference_ = −0.10 min *SD* = 0.25 min, 95% CI [−0.16, −0.03], *t*(56) = −2.85, *p* = .006). However, the Wilcoxon signed-rank test indicated that articulation rate did not significantly differ between noise and silence (*M*_difference_ = –0.68 wpm, *SD* = 13.18 wpm, 95% CI [−4.18, 2.81], *p* = .21). Speech rate (number of words divided by duration of recording) also did not significantly differ between noise and silence, but this analysis was less appropriate given the difference in number of unfilled pauses between noise and silence.

## Discussion

Despite the prevalence of background noise in everyday language contexts, research examining the effect of noise on speech production has been dominated by studies of acoustic-related changes in production. Very little work has investigated how noise affects higher-level properties of language such as syntax (but see Hanke, 2014; Harmon et al., 2021; Kemper et al., 2003). Moreover, moving beyond the descriptive level, we have lacked a satisfying account of why higher-level changes might be observed. In response to these gaps, the present study examined the effect of noise on the syntactic complexity of speech production during a picture description task and asked whether differences between speaking conditions were related to individual differences in cognitive control, as measured by the AX-CPT, Flanker, and Stroop tasks.

### Speakers alter their speech production in noise

Overall, we found clear differences between speech produced in noise relative to silence. At the acoustic level, we observed that participants spoke louder in noise, which was expected given previous research examining the effect of noise on the intensity of speech (e.g., Junqua, 1993). Speakers also produced fewer T-units, clauses and words when speaking in noise. Speaking in noisy environments is cognitively demanding, requiring focused attention on the act of speaking (Kemper et al., 2003), so it is unsurprising that participants would speak less overall.

Other findings were more surprising. Given that previous research has associated the production of mazes with utterance planning difficulty (Peach, 2013), we expected a greater number of mazes in noise. Contrary to our prediction, we found that participants produced fewer mazes in noise. One possibility is that participants are considering the needs of their listener during the picture description task, producing fewer mazes (and T-units, clauses, and words) to facilitate comprehension in noisy environments. This interpretation would accord with a substantial literature on perspective-taking (and, relatedly, audience design), the process by which individuals take into account what information their speech partner does and does not know during conversation (see Brown-Schmidt & Heller, 2018). Although ample evidence indicates that speakers’ productions are, to some extent, listener-driven (e.g., Brennan & Clark, 1996; Galati & Brennan, 2010; Gann & Barr, 2014), there is a longstanding debate regarding the circumstances that trigger listener-driven versus egocentric speaker-driven production processes. Vogels et al. (2020) reported that for situations where only the listener was exposed to increased cognitive load, speakers did not modify their productions to accommodate their listener unless the speaker themself had prior experience dealing with the same challenging task in which the listener was engaged. They suggested that listener-oriented modifications may only emerge when speakers are made aware that modifications could be beneficial—a suggestion which is supported by the present study, in which speakers were acutely aware of the potential cognitive burden noise presented to the listener.

It is also important to note that participants also produced fewer filled pauses in the noise condition, which seems to contradict the idea that participants were considering their listener during the task. A distinction has been drawn between filled pauses and unfilled pauses, with filled pauses argued to be listener-oriented (e.g., Clark & Fox Tree, 2002) and unfilled pauses argued to be speaker-oriented (e.g., Goldman-Eisler, 1968). Differences in the production of speaker- and listener-oriented disfluencies have been studied in individuals with autism spectrum disorder (ASD), who have been argued to exhibit less cognitive control (e.g., Solomon et al., 2008) and who are typically considered to be more self-centric speakers (e.g., Begeer et al., 2012). Individuals with ASD reportedly produce more unfilled pauses and fewer filled pauses than their typically developing peers (Lake et al., 2011; Shriberg et al., 2001 but see Engelhardt et al., 2017). Given these findings, it is plausible that noise would differentially affect the production of filled and unfilled pauses. If participants are considering their listener during production, we would expect them to produce more filled pauses and fewer unfilled pauses when speaking in noise, yet we observed that they produced a fewer number of both pause types. One possibility is that the presence of background noise may have led to reductions in syntactic complexity that was not captured through analyses of MLTU and CD. If participants did produce simpler structures, which are presumably easier to plan, when speaking in noise, this could explain the observed reduction in filled pauses. A further consideration is that, although speakers reduced the *number* of unfilled pauses in noise, the average *duration* of unfilled pauses increased. Under the account that unfilled pauses are typically speaker-oriented, increased unfilled pause duration in noise could potentially benefit the speaker by giving them more time to plan their utterances.

So far, we have provided evidence that speakers alter their speech in the face of noise. In addition to changes in vocal intensity, participants altered other (non-acoustic) characteristics of their speech. Some of these findings were anticipated—participants produced fewer T-units, clauses and words in noise. Others were unexpected—participants produced fewer disfluencies (i.e., filled pauses, unfilled pauses, and mazes) in noise. In other words, speech produced in noise is more concise and less disfluent, suggesting that speakers alter their speech to facilitate comprehension for their listener. However, closer inspection of listener-oriented filled pauses contradicts this interpretation; speakers produced fewer filled pauses in noise when we would expect them to produce more. To shed more light on these apparently contradictory findings, we turn to our measures of cognitive control.

### Cognitive control modulates speech production in noise

Previous work has shown that cognitive control is involved in various aspects of production ranging from lexical selection (e.g., Crowther & Martin, 2014; Maxfield, 2009, 2020; Shao et al., 2014) to fluency of language production (e.g., Engelhardt et al., 2010, 2013) to code-switching in bilingual speakers (e.g., Abutalebi & Green, 2008; Bosma & Blom, 2019; Yim & Bialystok, 2012). However, little is known about the role of cognitive control in underpinning production under adverse speaking conditions. Although participants in the current study experienced inference effects for each of our cognitive control tasks, significant correlations were observed only when analysing the Stroop data, potentially due to the more linguistic nature of the Stroop task compared with AX-CPT and the Flanker. We also acknowledge that, after participant exclusions, the AX-CPT and Flanker tasks were somewhat underpowered, which could also explain why significant correlations were only observed for the Stroop task.

Individuals who exhibited greater Stroop interference effects generally produced fewer clauses, words, and unfilled pauses in the noise relative to the silence condition, whereas those exhibiting smaller Stroop interference effects generally did not change their production when switching between the two conditions. If participants in the study were altering their speech solely for the benefit of the listener, it is counterintuitive that those with weaker cognitive control were the ones producing fewer clauses, words, and unfilled pauses in noise. This evidence suggests that participants were altering speech for their own benefit (i.e., to reduce their own cognitive burden against a distracting background) as well as for their listener’s benefit (i.e., to facilitate comprehension). However, given the weak-to-moderate reliability of Stroop interference scores observed here, it will be important to consider, in future work, whether speaker-oriented modifications are related to alternative measures of susceptibility to linguistic interference.

As introduced above, the challenge of speaking in noise is twofold. Not only is noise distracting for the speaker, but it also creates an adverse listening condition for the listener. When speaking in noise, speakers reduced the quantity of speech they produced as well as the number of unfilled pauses, filled pauses, and mazes (while increasing the duration of unfilled pauses). In addition, individuals with less cognitive control produced fewer clauses, words, and unfilled pauses in noise. Taken together, these findings suggest that there are likely both speaker- and listener-oriented modifications that occur when speaking in noise. Speaking in the presence of background noise entails the inhibition of distracting auditory input, so speakers may have reduced the quantity of speech and the number of unfilled pauses as they grappled with these demanding conditions. Correspondingly, we found that individuals with weaker cognitive control tended to produce fewer clauses, words, and unfilled pauses in noise. Although participants produced fewer unfilled pauses in noise, when they did produce unfilled pauses, the average duration of unfilled pauses was greater in noise compared with silence, which was not correlated with cognitive control. Because noise also presents a challenge for the listener, speakers may produce longer unfilled pauses to allow listeners more time to process particularly difficult utterances. In addition, participants may have also reduced the number of mazes (i.e., abandoned words/utterances, false starts/reformulations, and repetitions) for their listener’s benefit as a means of facilitating comprehension. If it is the case that filled pauses assist listeners in the comprehension of complex syntactic structures (Bailey & Ferreira, 2003; Watanabe et al., 2008), the reduction of filled pauses in noise may be reflective of reduced complexity of speech produced in noise. Alternatively, the reduction of filled pauses may be a method of streamlining speech for the listener. Neither reductions in mazes nor filled pauses were significantly associated with cognitive control. This association was also not observed for number of T-units, although the effect of noise versus silence on this measure tended to be less stable overall (Section 1, Supplemental Materials).

### Points of convergence and divergence with previous studies

As mentioned above, speech production in noise (beyond the acoustic level) is an under-studied area, however, previous work by Kemper et al. (2003) and Harmon et al. (2021) provided evidence that speakers alter certain higher-level properties of their speech due to noise. Kemper et al. (2003) were primarily interested in age-related differences in speech production while completing concurrent tasks, whereas Harmon et al. (2021) were interested in how different types of background noise affect speech production. In both studies, participants completed an elicitation task in which they answered prompts while speaking in noise. Most relevant to the current study, Harmon et al. (2021) similarly reported fewer unfilled pauses in noise. They proposed two potential explanations for these surprising results: speakers produced fewer unfilled pauses to maintain the listener’s focus in the face of distraction or to maintain their own focus on the task. Due to limitations on their experimental design, Harmon et al. (2021) were unable to determine whether the reduction in unfilled pauses served to maintain the speaker’s focus or the listener’s focus. In the present study, because we ensured both the speaker and listener were exposed to noise and collected measures of cognitive control, we were in a position to answer this question. Given that we found that individuals with less cognitive control produced fewer unfilled pauses in noise, our results suggest that speakers reduced the number of unfilled pauses in noise to maintain their own focus on speaking without becoming distracted by the background noise.

In addition, we observed that speakers reduced their overall quantity of speech when speaking in noise, which parallels Hadley et al.’s (2019) observation of shorter turn durations as noise levels increased. While our use of a picture description task allows for slightly more constrained production (at the semantic level) than the free conversations in Hadley et al. (2019), the picture description task still allows for freer production than more goal-oriented tasks (e.g., Diapix task, map task). Thus, participants may have adopted a strategy of producing less detailed descriptions in the more challenging speaking condition. Participants also produced longer unfilled pauses in noise, which mimics the longer inter-turn pauses reported by Aubanel et al. (2011) in noise. We found that increased average unfilled pause duration did not correlate with performance on the Stroop task, which leaves open the possibility that speakers pause for longer durations in noise to provide listeners more comprehension time.

Contrary to our findings, both Kemper et al. (2003) and Harmon et al. (2021; with the exception of unfilled pauses) reported increased disfluencies in noise while we observed decreased disfluencies. However, topic of conversation has been shown to influence the production of disfluencies (Bortfeld et al., 2001; Schachter et al., 1991), thus pronounced differences in speech elicitation techniques (open-ended questions versus a semantically controlled picture description task) may explain these diverging results. Most crucially, unlike Kemper et al. (2003) and Harmon et al. (2021), the current study was able to examine whether speakers may alter their speech for the benefit of their listener because both the participant and the experimenter were exposed to the background noise. Based on our findings, we propose that reduced disfluencies (specifically the reduction of filled pauses and mazes), are listener-oriented speech modifications. Both Kemper et al. (2003) and Harmon et al. (2021) were not able to examine the role of the listener on production, which may also account for differences in the disfluency results. Overall, our findings strongly suggest that speakers alter their speech not only due to their own exposure to the demands of background noise, but also due to the listener’s exposure to the noise, producing both speaker- and listener-oriented speech modifications. More specifically, we consider the reduction of clauses, words, and unfilled pauses to be speaker-oriented because they are associated with cognitive control.

### Limitations and future directions

One point of consideration for future work is the specificity of videoconferencing conversation relative to in-person conversation. Interestingly, Tracy et al. (2020) explored how acoustic properties of speech are affected by communication modality, reporting increased vocal intensity and self-reported levels of vocal effort for remote communication relative to in-person communication. Thus, considering remote communication is already effortful, it may be that the additional burden of noise coupled with online nature of our study created a speaking condition that disfavoured the production of more complex structures. This extra layer of burden could explain why CD and MLTU did not differ between noise and silence conditions, a finding that sits in contrast to portions of the existing literature. Under less effortful, in-person conditions, greater differences in complexity may emerge. Nevertheless, although we acknowledge that remote language production differs from in-person language production, we stress that the generalisability of our findings is bolstered by the replication of well-supported acoustic effects (e.g., increased intensity in noise) in our data.

Beyond examining complexity differences between noise and silence, we also sought to differentiate between speaker- and listener-oriented modifications based on correlations between complexity differences and cognitive control. Future work could more directly assess which modifications are speaker- or listener-oriented by manipulating which member of the dyad is exposed to noise (e.g., speaker-only, listener-only). We note that the picture description task was not meant to foster conversation between the speaker and listener because the experimenter did not communicate with the participant as they were describing the images. Although participants were encouraged to consider the needs of their listener throughout the task, there was not necessarily a task-related incentive to do so. As such, future work should consider the use of goal-oriented tasks (e.g., Diapix task, map task) that require collaboration for successful task completion.

## Conclusion

The present study examined whether (1) background noise affects the syntactic complexity of speech production; and (2) cognitive control predicted noise-induced differences in complexity. First, we found compelling evidence that speakers alter the way they speak in noise versus silence. While we acknowledge the value in conducting this study under volume-controlled, in-person conditions, our results suggest that speakers take into account the needs of their listener when speaking in noisy environments while also altering speech for their own benefit. We found evidence that speakers produced fewer mazes and filled pauses in noise, potentially indicative of speakers streamlining their speech for the sake of their listener. Speakers also produced fewer T-units, clauses, words, and unfilled pauses, which may be indicative of speakers simplifying speech to reduce their own cognitive burden, although it is important to consider that average unfilled pause duration was actually increased in noise. Most notably, participants with lower cognitive control, as indexed by larger Stroop RT interference effects, produced fewer clauses, words, and unfilled pauses in noise, providing further support that these reductions were for the speaker’s benefit. To further examine the distinction between speaker- and listener-oriented speech modifications, future analyses will consider more qualitative assessments of the production data, examining the overall quality of the descriptions produced in noise versus silence and potential differences in the types of syntactic structures produced in each condition.

## Supplemental Material

sj-docx-1-qjp-10.1177_17470218221124869 – Supplemental material for Noise-induced differences in the complexity of spoken language Click here for additional data file. Supplemental material, sj-docx-1-qjp-10.1177_17470218221124869 for Noise-induced differences in the complexity of spoken language by Catherine T Pham and Elisabeth A Karuza in Quarterly Journal of Experimental Psychology

## Notes

1 We note that our error analyses include instances of existential *there* disagreement (e.g., ***there is hills*** *in the background*), which may not necessarily be considered ungrammatical by participants (see Krejci & Hilton, 2017). Consequently, error analyses were also run excluding existential *there* disagreement, and number of errors still did not significantly differ between noise and silence.
