# Supplementary material for: Noise-induced differences in the complexity of spoken language
Source: Q J Exp Psychol (Hove). 2022 Oct 6;76(7):1609–31. doi: 10.1177/17470218221124869 (PMC10280673; doi:10.1177/17470218221124869)
Supplement: sj-docx-1-qjp-10.1177_17470218221124869 – Supplemental material for Noise-induced differences in the complexity of spoken language [file sj-docx-1-qjp-10.1177_17470218221124869.docx]

Supplementary Material for:

**Noise-Induced Differences in the Complexity of Spoken Language**

Catherine T. Pham and Elisabeth A. Karuza

Department of Psychology, The Pennsylvania State University, University Park, PA 16802

**Section 1**

**Method of Model Building**

For the linear mixed effects models presented in Tables S1 and S3, random effects were determined by beginning with the fullest structure justified by the experimental design and trimming by-item (Picture), then by-participant slopes for Run and Condition until convergence without singular fit was achieved. Because the effect of Condition (noise *versus* silence) was our primary fixed effect of interest, and participant variances tend to exceed item variances (Jaeger, 2010), we prioritized inclusion of a by-participant random slope for Condition whenever possible. Once convergence without singular fit was achieved through this trimming method, model comparisons then confirmed that the inclusion of further random effects was not warranted. R code for our full process of model selection is provided here: <https://pennstateoffice365-my.sharepoint.com/:u:/g/personal/ctp49_psu_edu/ESGJHs8uU4NFo-2_qLmbZ8IBg98fAu59HJuovYE8bcC-lA?e=nmNiBS>. For comparison purposes, we also include code for random intercepts-only versions of each of the models presented in Table A1. With the exception of Model 3 (T-units), significant main effects of Condition were stable when this sparser random effect structure was used.

Models were assessed through diagnostic residual plots. In cases where the residuals were a potential concern, either 1) a log-transformation was applied (Mean Length of T-unit, Clausal Density, Total Pause Duration), or 2) in cases of lower count variables (Mazes, Errors, Omissions, Lexical Fillers), models were refit using a generalized linear mixed model (*glmer*, family = poisson). With the exception of Total Unfilled Pause Duration, for which a significant main effect of Condition was observed following log-transformation (β = 0.03, *t* = 2.76, *p* = 0.008), the pattern of significant results presented in the tables below was unchanged.

#### Full Summary of Statistical Analyses

Table S1. Coefficients and corresponding *t*-values and *p*-values for each predictor in a linear mixed effects model examining all main effect and interactions of Condition and Run on eleven measures of complexity. Significant values are bolded.

| **Predictor** | **Coefficient** | ***T*-value** | ***P*-value** |
| --- | --- | --- | --- |
| **Ratio-based Measures** |  |  |  |
| MODEL 1: *Mean Length of T-unit* |  |  |  |
| Condition (Noise vs. Silence) | 0.10 | 0.99 | 0.32 |
| Run | 0.09 | 0.91 | 0.37 |
| Condition (Noise vs. Silence)*Run | –0.05 | –0.59 | 0.56 |
| MODEL 2: *Clausal Density* |  |  |  |
| Condition (Noise vs. Silence) | 0.02 | 1.53 | 0.13 |
| Run | 0.01 | 0.45 | 0.65 |
| Condition (Noise vs. Silence)*Run | –0.01 | –0.45 | 0.65 |
| **Measures of Quantity** |  |  |  |
| MODEL 3: *Number of T-units* |  |  |  |
| **Condition (Noise vs. Silence)** | **0.70** | **2.19** | **0.03** |
| Run | 0.46 | 1.29 | 0.20 |
| Condition (Noise vs. Silence)*Run | 0.36 | 1.25 | 0.22 |
| MODEL 4: *Number of Clauses* |  |  |  |
| **Condition (Noise vs. Silence)** | **1.49** | **3.22** | **0.002** |
| Run | 1.37 | 2.07 | 0.096 |
| Condition (Noise vs. Silence)*Run | 0.25 | 0.62 | 0.54 |
| MODEL 5: *Number of Words* |  |  |  |
| **Condition (Noise vs. Silence)** | **9.53** | **2.98** | **0.004** |
| **Run** | **9.07** | **2.34** | **0.02** |
| Condition(Noise vs. Silence)*Run | 1.76 | 0.69 | 0.49 |
| **Measures of Disfluencies and Errors** |  |  |  |
| MODEL 6: *Number of Unfilled Pauses* |  |  |  |
| **Condition (Noise vs. Silence)** | **2.60** | **4.33** | **<0.0001** |
| **Run** | **1.34** | **2.49** | **0.02** |
| Condition (Noise vs. Silence)*Run | 0.57 | 1.66 | 0.10 |
| MODEL 7A^[[1]](#footnote-1)^: *Total Unfilled Pause Duration* |  |  |  |
| Condition (Noise vs. Silence) | 0.74 | 1.63 | 0.11 |
| Run | 0.31 | ­0.82 | 0.41 |
| Condition (Noise vs. Silence)*Run | –0.007 | -0.02 | 0.99 |
| MODEL 7B: *Average Unfilled Pause Duration* |  |  |  |
| **Condition (Noise vs. Silence)** | **–12.08** | **–2.27** | **0.03** |
| Run | –6.77 | ­–0.84 | 0.48 |
| Condition (Noise vs. Silence)*Run | –5.72 | –1.19 | 0.24 |
| MODEL 8: *Number of Filled Pauses* |  |  |  |
| **Condition (Noise vs. Silence)** | **0.79** | **3.35** | **<0.001** |
| Run | 0.25 | 1.20 | 0.23 |
| **Condition (Noise vs. Silence)*Run** | **0.64** | **2.72** | **0.007** |
| MODEL 9: *Number of Mazes* |  |  |  |
| **Condition (Noise vs. Silence)** | **0.62** | **2.50** | **0.01** |
| Run | 0.23 | 1.04 | 0.30 |
| Condition (Noise vs. Silence)*Run | 0.13 | 0.53 | 0.60 |
| MODEL 10: *Number of Errors* |  |  |  |
| Condition (Noise vs. Silence) | 0.03 | 0.17 | 0.87 |
| Run | 0.13 | 1.14 | 0.26 |
| Condition (Noise vs. Silence)*Run | 0.06 | 0.52 | 0.61 |
| MODEL 11: *Number of Omitted Words* |  |  |  |
| Condition (Noise vs. Silence) | 0.03 | 0.20 | 0.84 |
| **Run** | **0.22** | **2.14** | **0.03** |
| Condition (Noise vs. Silence)*Run | –0.002 | –0.01 | 0.99 |

Table S2. Correlation coefficients and *p*-values for the correlation analyses between Silence–Noise differences for each complexity measure and cognitive control measure. Significant values are bolded.

| **Cognitive Control Measure** | **Complexity Measure** | **Correlation Coefficient** | ***P*-value** |
| --- | --- | --- | --- |
| AX-CPT  Behavioral Shift Index | Number of T-units | 0.07 | 0.62 |
|  | Number of Clauses | 0.16 | 0.27 |
|  | Number of Words | 0.21 | 0.13 |
|  | Number of Unfilled Pauses | –0.007 | 0.96 |
|  | Average Unfilled Pause Duration | –0.01 | 0.92 |
|  | Number of Filled Pauses | 0.04 | 0.79 |
|  | Number of Mazes | –0.04 | 0.78 |
| Flanker  RT Interference | Number of T-units | –0.13 | 0.39 |
|  | Number of Clauses | –0.06 | 0.70 |
|  | Number of Words | –0.09 | 0.57 |
|  | Number of Unfilled Pauses | 0.13 | 0.41 |
|  | Average Unfilled Pause Duration | 0.06 | 0.70 |
|  | Number of Filled Pauses | 0.09 | 0.57 |
|  | Number of Mazes | –0.23 | 0.14 |
| Stroop  RT Interference | Number of T-units | –0.13 | 0.33 |
|  | **Number of Clauses** | **–0.35** | **0.008** |
|  | **Number of Words** | **–0.31** | **0.02** |
|  | **Number of Unfilled Pauses** | **–0.31** | **0.02** |
|  | Average Unfilled Pause Duration | 0.17 | 0.19 |
|  | Number of Filled Pauses | 0.22 | 0.10 |
|  | Number of Mazes | 0.002 | 0.99 |

Table S3. Coefficients and corresponding *t*-values and *p*-values for each predictor in a linear mixed effects model examining all main effect and interactions of Condition and Run on post-hoc analyses of hedges and lexical fillers. Significant values are bolded.

| **Predictor** | **Coefficient** | ***T*-value** | ***P*-value** |
| --- | --- | --- | --- |
| MODEL 1: *Number of Hedges* |  |  |  |
| Condition (Noise vs. Silence) | 0.41 | 1.83 | 0.07 |
| Run | –0.18 | –0.91 | 0.36 |
| Condition (Noise vs. Silence)*Run | 0.09 | 0.43 | 0.67 |
| MODEL 2: *Number of Lexical Fillers* |  |  |  |
| Condition (Noise vs. Silence) | 0.30 | 1.65 | 0.10 |
| Run | 0.37 | 1.73 | 0.09 |
| Condition (Noise vs. Silence)*Run | –0.28 | –1.67 | 0.10 |

**Section 2**

**List of Hedges**

Relational hedges that were included in the hedge analyses are presented below. A subset of these were adopted from Ulinski and Hirschberg (2019). Adverbs that appear in parentheses indicate that the relational phrase can optionally include the adverb and both were included in our analyses. Also note that all verb forms were accounted for in the analyses (e.g., past and present tense) as well as contractions and their non-contracted counterparts (e.g., *I’d* and *I would*).

- according to
- appear
- arguably
- assume
- believe
- consider
- could
- doubt
- estimate
- expect
- feel
- find
- guess
- hear
- I am (just) going to say
- I am not (exactly/really/too) sure
- I can’t (really) tell
- I don’t know
- I mean
- I would (just) conclude
- I would go
- I would say
- imagine
- impression
- in my mind
- in my opinion
- in my understanding
- in my view
- know
- likely
- look
- may
- maybe
- might
- my thinking
- my understanding
- necessarily
- perhaps
- possibly
- presume
- presumably
- probably
- seem
- seemingly
- should
- sound
- speculate
- suggest
- suppose
- sure
- tend
- think
- understand
- unlikely
- unsure

#### Section 3

#### Transcription Sample

The transcription below is taken from a description of the beach scene image in silence. Each T-unit is numbered. The code designating each clause type has been inserted at the end of each T-unit in brackets. All words enclosed in parentheses were removed prior to calculating MLTU, CD, and number of T-Units, clauses, and words.

1. it looks like they're (pause: 1200 ms) sort of on a beach [MC] [NOM]
2. but (there’s gra#) it looks like there’s grass on the left side with (a house#) (pause: 929 ms) almost !like a little beach house with a garage and !like two windows on the side and then one window and then one door (^pause: 892 ms) [MC] [NOM]
3. it’s !like a one story house [MC]
4. there’s a mountain in the background (pause: 408 ms) or !like a (pause: 920 ms) higher landmark (on the#) in the background [MC]
5. there’s a guy (^pause: 669 ms) flying a kite (^pause: 1812 ms) [MC] [PRT]
6. and (i#) $there’s clouds in the sky [MC]
7. ($/it) doesn’t look like there’s any sun (^pause: 854 ms) [MC] [NOM]
8. (there#) (pause: 585 ms) in the bottom left_hand corner (there lo#) there looks like a soccer ball [MC]
9. but @I don’t know (^pause: 1069 ms) why it’s just by itself just rolling away it looks like it (^pause: 1277 ms) [MC] [NOM] [PRT]
10. and then above it (pause: 576 ms) there’s a (pause: 320 ms) girl petting a sea_lion (^pause: 1031 ms) [MC] [PRT]
11. and she’s underneath an umbrella (pause: 558 ms) on a towel [MC]
12. she’s in a dress and looks like (pause: 567 ms) just slides for shoes [MC]
13. she has short hair (^pause: 1031 ms) [MC]
14. she’s (pause: 1421 ms) getting a drink from (pause: 622 ms) a younger (pause: 1050 ms) it looks like (pause: 389 ms) (&like) a boy (^pause: 508 ms) [MC]
15. and he has !like a (beach_ball#) (pause: 313 ms) beach volleyball I think in his hand or a beach_ball (^pause: 799 ms) [MC]
16. he has short hair as well [MC]
17. and he looks like he has !like tennis_shoes on (^pause: 863 ms) [MC] [NOM]
18. and (pause: 1523 ms) (UH) in about the middle (pause: 520 ms) bottom portion of the picture (there’s another#) there’s two people that look like they're (pause: 567 ms) building sandcastles [MC] [REL] [NOM]
19. but they look like they’re arguing (&arguing) on (^pause: 864 ms) where everything goes (^pause: 863 ms) [MC] [NOM] [NOM]
20. the one on the right’s a little bit younger [MC]
21. and (pause: 1180 ms) they’re wearing a hat with a (pause: 455 ms) dress and it looks like leggings (^pause: 399 ms) [MC]
22. and the other one has dreadlocks with a tank_top and shorts (^pause: 754 ms) [MC]
23. and there are seashells around them (^pause: 2331 ms) [MC]
24. (UH) (pause: 687 ms) and then to the right (pause: 437 ms) there’s another person walking with (a#) (pause: 360 ms) it looks like a pail or a bucket (^pause: 1515 ms) [MC] [PRT]
25. and their hair is (pause: 647 ms) down but tied up a little bit (pause: 719 ms) in the back [MC]
26. and they have (pause: 913 ms) (UH short sl#) (pause: 483 ms) a t_shirt and shorts (^pause: 879 ms) [MC]
27. and it looks like when you get closer to the water there might be more rocks (^pause: 399 ms) cause there might be !like a pathway before it reaches the water (^pause: 949 ms) [MC] [ADV] [NOM] [ADV] [ADV]
28. and (pause: 727 ms) there’s a mermaid (pause: 550 ms) on a rock [MC]
29. and she looks like she’s either drinking something or she’s gonna play something do something with her mouth (^pause: 727 ms) [MC] [NOM] [NOM] [INF] [INF]
30. and she has it looks like a t_shirt on (^pause: 647 ms) as well [MC]
31. and then there’s a (little#) (pause: 951 ms) !like little sailboat in the water [MC]
32. but it looks like (^pause: 879 ms) someone !like made it out of !like paper (^pause: 442 ms) [MC] [NOM]
33. it’s not a life_size (pause: 951 ms) (UM) (pause: 790 ms) boat (^pause: 514 ms) [MC]
34. and then to the right of that (there’s a#) (pause: 381 ms) it looks like a (&like a) sea_monster (^pause: 835 ms) [MC]
35. it looks like a serpent in a way (pause: 645 ms) with weird eyes and pointy teeth (^pause: 642 ms) [MC]
36. and it’s very long [MC]
37. and (pause: 1178 ms) besides that and the little sailboat there’s nothing really else in the water [MC]
38. there’s the (pause: 374 ms) mermaid [MC]
39. ($/it) looks like she’s about to go into the water (^pause: 796 ms) [MC] [NOM] [INF]
40. but she’s just sitting there (pause: 265 ms) on the rock [MC]

**Section 4**

**Supplemental Reliability Analyses**

One question we explore in the current study is the extent to which individual differences in cognitive control predict differences in complexity between speech produced in noise *versus* silence. To address this question, we performed correlational analyses between mean Stroop RT interference scores (incongruent–neutral) and Noise-Silence difference scores for a subset of our measures of complexity. Given concerns regarding the use of difference scores in this context (Draheim et al., 2019), the reliability for each complexity measure was assessed using standard split-half reliability and then applying Spearman-Brown’s prophecy formula to adjust correlations to reflect the full length of the test. For completeness, we report reliability for all complexity measures. (Table S4).

Table S4. Reliability coefficients and 95% CIs for each complexity measure for each condition (Noise and Silence) and difference scores (Noise–Silence). All coefficients and CIs were Spearman-Brown adjusted with the exception of difference scores for MLTU, mazes, and errors. Difference scores for MLTU, mazes, and errors resulted in negative reliability coefficients, in which case the Spearman-Brown adjustment was not applied. Parsons et al. (2019) note that negative reliability estimates can occur when examining difference scores, in which case they recommend reporting the negative reliability coefficient but interpreting it as a reliability of 0.

| **Complexity Measure** | **Noise** | | **Silence** | | **Difference Scores** | |
| --- | --- | --- | --- | --- | --- | --- |
|  | ***coefficient*** | ***95% CI*** | ***coefficient*** | ***95% CI*** | ***coefficient*** | ***95% CI*** |
| MLTU | 0.74 | [0.55, 0.84] | 0.68 | [0.45, 0.81] | –0.20 | [–0.44, 0.06] |
| CD | 0.47 | [0.09, 0.69] | 0.69 | [0.47, 0.82] | 0.17 | [–0.41, 0.52] |
| T-units | 0.83 | [0.72, 0.90] | 0.79 | [0.64, 0.88] | 0.16 | [–0.43, 0.51] |
| Clauses | 0.81 | [0.69, 0.89] | 0.83 | [0.70, 0.89] | 0.23 | [–0.31, 0.55] |
| Words | 0.88 | [0.79, 0.93] | 0.84 | [0.72, 0.90] | 0.38 | [–0.06, 0.63] |
| Unfilled Pauses | 0.86 | [0.76, 0.92] | 0.84 | [0.73, 0.91] | 0.69 | [0.47, 0.82] |
| Average Unfilled Pause Duration | 0.84 | [0.74, 0.91] | 0.82 | [0.69, 0.89] | –0.64 | [–1.80, 0.04] |
| Filled Pauses | 0.94 | [0.89, 0.96] | 0.93 | [0.88, 0.96] | 0.22 | [–0.32, 0.55] |
| Mazes | 0.64 | [0.39, 0.79] | 0.66 | [0.42, 0.80] | –0.02 | [–0.28, 0.25] |
| Errors | 0.45 | [0.06, 0.68] | 0.70 | [0.49, 0.82] | –0.03 | [–0.28, 0.24] |
| Omissions | 0.80 | [0.65, 0.88] | 0.81 | [0.68, 0.89] | 0.41 | [–0.01, 0.65] |

While the reliability of each condition considered separately is generally high, the reliability of the Noise–Silence difference scores are rather low. Thus, as reported in the main text, simple correlational analyses were supplemented with additional analyses that did not require use of a mean subtraction methodology for the complexity metrics (i.e., a hierarchical modeling approach; see Rouder & Haaf, 2019). This approach addresses some concerns related to use of Noise-Silence difference scores, but use of the Stroop RT interference scores was still a consideration due to their weak-to-moderate reliability (0.46). In particular, Draheim et al. (2019) highlight concerns surrounding the use of RT differences, especially when considering contaminating effects due to the speed-accuracy trade-off. They suggest a more suitable alternative to RT differences is the use of accuracy-based measures of individual differences. Therefore, we conducted correlation analyses between Stroop performance and differences in complexity using overall Stroop accuracy scores as our predictor variable (Table S5). However, results revealed no significant correlations.

Table S5. Correlation coefficients and *p*-values for the correlation analyses between Noise–Silence differences for each complexity measure and overall accuracy scores on the Stroop task. All correlations were not significant.

| **Complexity Measure** | **Correlation Coefficient** | ***P*-value** |
| --- | --- | --- |
| Number of T-units | –0.20 | 0.13 |
| Number of Clauses | –0.06 | 0.66 |
| Number of Words | –0.06 | 0.66 |
| Number of Unfilled Pauses | –0.04 | 0.77 |
| Average Unfilled Pause Duration | 0.24 | 0.07 |
| Number of Filled Pauses | 0.17 | 0.21 |
| Number of Mazes | 0.02 | 0.91 |

1. We did not include analyses of total unfilled pause duration in the main manuscript because total duration cannot disentangle few long pauses from many short pauses. That analysis was replaced with MODEL 7B. [↑](#footnote-ref-1)
